# A domain-oriented approach to the reduction of combinatorial complexity in signal transduction networks

**DOI:** 10.1186/1471-2105-7-34

**Published:** 2006-01-23

**Authors:** Holger Conzelmann, Julio Saez-Rodriguez, Thomas Sauter, Boris N Kholodenko, Ernst D Gilles

**Affiliations:** 1 Institute for System Dynamics and Control Engineering, University of Stuttgart, Pfaffenwaldring 9, 70569 Stuttgart, Germany; 2 Max-Planck-lnstitute for Dynamics of Complex Technical Systems, Sandtorstr. 1, 39106 Magdeburg, Germany; 3 Department of Pathology, Anatomy and Cell Biology, Thomas Jefferson University, 1020 Locust St., Philadelphia, PA 19107, USA

## Abstract

**Background:**

Receptors and scaffold proteins possess a number of distinct domains and bind multiple partners. A common problem in modeling signaling systems arises from a combinatorial explosion of different states generated by feasible molecular species. The number of possible species grows exponentially with the number of different docking sites and can easily reach several millions. Models accounting for this combinatorial variety become impractical for many applications.

**Results:**

Our results show that under realistic assumptions on domain interactions, the dynamics of signaling pathways can be exactly described by reduced, hierarchically structured models. The method presented here provides a rigorous way to model a large class of signaling networks using macro-states (macroscopic quantities such as the levels of occupancy of the binding domains) instead of micro-states (concentrations of individual species). The method is described using generic multidomain proteins and is applied to the molecule LAT.

**Conclusion:**

The presented method is a systematic and powerful tool to derive reduced model structures describing the dynamics of multiprotein complex formation accurately.

## Background

Receptor-mediated signal transduction is the subject of intense research since it plays a crucial role in the regulation of a variety of cellular functions. The ligand binding to a receptor triggers conformational changes that allow for receptor dimerization and phosphorylation of numerous residues. The subsequent formation of multiprotein signaling complexes on these receptors and their scaffolding adaptor proteins initiates a variety of signaling pathways. The number of feasible different multiprotein species grows exponentially with the number of binding domains, and can easily reach thousands or even millions [1,2]. In the past years, a large number of mathematical models have attempted to describe signaling phenomena and to get deeper insights into the dynamics of cellular responses [3-11]. Most of these models did not consider the combinatorial variety of all possible species and interactions [1,2]. The obvious advantage of models neglecting the combinatorial complexity is there are less ordinary differential equations (ODEs) that have to be considered. For example, if all possible species were included in the model of Schoeberl et al. [6], the number of variables would grow from around 100 to almost 40000 [2]. However, there is no general method to decide a priori which species play a crucial role and which can be neglected. Recent investigations indicate that the structure of reduced models which focus on the most important species, is highly dependent on the kinetic parameters [12]. The procedure described in [12] is based on deleting species from a full network model to find the smallest network that reproduces the dynamics of a set of observed quantities.

In 1998, the stochastic simulation tool StochSim was developed to handle the problem of combinatorial complexity [11,13]. The number of multi-state protein complexes and chemical reactions could reach millions in deterministic models of bacterial chemotaxis, and StochSim was employed to circumvent this problem [11]. The use of StochSim is especially practical for networks in small volumes with low numbers of molecules, where a stochastic algorithm more closely describes the physical reality than a deterministic algorithm. For large networks with hundreds of different proteins the computational cost would be extremly high increasing proportionally to the number of molecules squared. An alternative deterministic approach considers a complete mechanistic description that accounts for all possible species and reactions [1,14]. In the software tool BioNetGen, an algorithm for rule-based modeling is implemented [15]. However, the computational cost also appears to be very high, making it difficult to analyze large networks with a vast number of states.

Recently, a new approach has been introduced where a mechanistic picture of all possible states is substituted by a macro-description that follows the occupancy levels and other characteristics of individual domains, e.g. the phosphorylation states of these sites [16]. These quantitative indicators of the system (levels of occupancy) are referred to as macro-states. A modeling description using macro-states accounts for limitations in the current techniques to measure concentrations of individual multiprotein species. The results of common biological measurements (e.g. immunoprecipitation followed by western blotting) correspond to cumulative quantities like levels of occupancy or degrees of phoshorylation. Thus, the introduction of these and similar quantities into modeling facilitates the comparison of model variables with experimental readouts. This approach adopts the point of view of Pawson and Nash [17] that molecular domains instead of molecules are the fundamental elements of signal transduction. In their review they discuss that the regulation of many different cellular processes and functions require the use of protein interaction domains, and that aberrant interactions can induce abnormal cellular behaviour and diseases. We show that besides these considerations this macroscopic description also provides a number of mathematical benefits. In their work Borisov et al. demonstrate that for scaffold proteins with independent binding sites and scaffolds with one controling domain, the dynamics of these macroscopic states can be accurately described by reduced models [16]. However, a methodology to derive the reduced model equations for any scaffold with a more complex pattern of domain interactions is still missing. In this contribution, we will introduce a new systematic approach formalizing and extending the model reduction presented in [16]. Our method not only answers the questions whether a mathematically accurate model reduction is possible in a given system, but also how many and which equations are required. Our approach is based on a state space transformation linking the approaches provided in [15] and [16]. This link is achieved by a hierarchical structure of the state space transformation introducing mesoscopic state variables (describing different levels of detail) in addition to macroscopic and microscopic states defined in [16]. In contrast to many other model reduction methods our approach is independent of exact numerical values. Often, only qualitative biological knowledge about domain interactions is needed to derive the reduced model equations. For instance, data reported for receptor tyrosine kinases provide qualitative knowledge about domain interactions [18]. The ligand binding to these receptors controls their dimerization and the rate of phosphorylation of docking sites, which bind multiple partners. Here, we address the question of how different domain interactions influence the structure of a reduced model and how many states are required in order to describe the system dynamics accurately. We demonstrate which signaling systems can be described using only macro-states, and which additionally require the consideration of mesoscopic states.

The contribution is structured as follows. First, we introduce our method using simple examples reconsidering some of the cases described in [16] in order to show that our method provides the exact same results. For example, if the binding sites are independent, or only one of the domains controls some others (which is the case of a scaffolding adaptor protein phosphorylated by receptor kinase, e.g., insulin receptor substrate phosphorylated by the insulin receptor [19]) it can be shown that the number of states can be strongly reduced. Additionally, we confirm our results via dynamical simulations and discuss the advantages of the reduced models. Second, we generalize these results and also consider an example with a more complex pattern of domain interactions, and finally, we apply the method to a real scaffold protein, namely the adaptor molecule LAT (Linker for activation of T-cells).

## Results

In order to introduce and discuss our method in an descriptive manner, we first want to introduce some definitions and outline the principles shortly. Afterwards, we will consider some simple generic examples (scaffold proteins with three and four binding domains) in more detail. Of course, these examples might also be treated without recourse to our theoretical developments, because the number of states is relatively small. But precisely because some of the results may appear intuitive, this shows the potential of our method which is also applicable to other more complicated problems. Additionally, we generalize two important cases to scaffolds with n binding sites (namely independent binding domains and domains that are controlled by one controlling site). We also provide the generalized transformation matrix for proteins with n binding sites and exemplify our method considering the scaffold molecule LAT (Linker of activation in T-cells).

### Definitions

In the following, we will consider a receptor or scaffold protein *R *with *n *distinct binding domains *i *(i.e *i *= 1, 2, ..., *n*). Each of these domains *i *can bind *m*_*i *_different effector proteins Eij
 MathType@MTEF@5@5@+=feaafiart1ev1aaatCvAUfKttLearuWrP9MDH5MBPbIqV92AaeXatLxBI9gBaebbnrfifHhDYfgasaacH8akY=wiFfYdH8Gipec8Eeeu0xXdbba9frFj0=OqFfea0dXdd9vqai=hGuQ8kuc9pgc9s8qqaq=dirpe0xb9q8qiLsFr0=vr0=vr0dc8meaabaqaciaacaGaaeqabaqabeGadaaakeaacqWGfbqrdaqhaaWcbaGaemyAaKgabaGaemOAaOgaaaaa@30A4@ with *j *= 1, ..., *m*_*i*_, and hence can be in *m*_*i *_+ 1 different states (unoccupied or occupied by one of the *m*_*i *_effectors). The number of feasible micro-states for such a scaffold protein is

∏i=1n(mi+1).     (1)
 MathType@MTEF@5@5@+=feaafiart1ev1aaatCvAUfKttLearuWrP9MDH5MBPbIqV92AaeXatLxBI9gBaebbnrfifHhDYfgasaacH8akY=wiFfYdH8Gipec8Eeeu0xXdbba9frFj0=OqFfea0dXdd9vqai=hGuQ8kuc9pgc9s8qqaq=dirpe0xb9q8qiLsFr0=vr0=vr0dc8meaabaqaciaacaGaaeqabaqabeGadaaakeaadaqeWbqaaiabcIcaOiabd2gaTnaaBaaaleaacqWGPbqAaeqaaOGaey4kaSIaeGymaeJaeiykaKIaeiOla4IaaCzcaiaaxMaadaqadaqaaiabigdaXaGaayjkaiaawMcaaaWcbaGaemyAaKMaeyypa0JaeGymaedabaGaemOBa4ganiabg+Givdaaaa@3EAC@

The number of possible reactions that have to be considered is

∑i=1nmi∏k=1,k≠in(mk+1).     (2)
 MathType@MTEF@5@5@+=feaafiart1ev1aaatCvAUfKttLearuWrP9MDH5MBPbIqV92AaeXatLxBI9gBaebbnrfifHhDYfgasaacH8akY=wiFfYdH8Gipec8Eeeu0xXdbba9frFj0=OqFfea0dXdd9vqai=hGuQ8kuc9pgc9s8qqaq=dirpe0xb9q8qiLsFr0=vr0=vr0dc8meaabaqaciaacaGaaeqabaqabeGadaaakeaadaaeWbqaaiabd2gaTnaaBaaaleaacqWGPbqAaeqaaaqaaiabdMgaPjabg2da9iabigdaXaqaaiabd6gaUbqdcqGHris5aOWaaebCaeaacqGGOaakcqWGTbqBdaWgaaWcbaGaem4AaSgabeaakiabgUcaRiabigdaXiabcMcaPiabc6caUaWcbaGaem4AaSMaeyypa0JaeGymaeJaeiilaWIaem4AaSMaeyiyIKRaemyAaKgabaGaemOBa4ganiabg+GivdGccaWLjaGaaCzcamaabmaabaGaeGOmaidacaGLOaGaayzkaaaaaa@4E02@

Eij
 MathType@MTEF@5@5@+=feaafiart1ev1aaatCvAUfKttLearuWrP9MDH5MBPbIqV92AaeXatLxBI9gBaebbnrfifHhDYfgasaacH8akY=wiFfYdH8Gipec8Eeeu0xXdbba9frFj0=OqFfea0dXdd9vqai=hGuQ8kuc9pgc9s8qqaq=dirpe0xb9q8qiLsFr0=vr0=vr0dc8meaabaqaciaacaGaaeqabaqabeGadaaakeaacqWGfbqrdaqhaaWcbaGaemyAaKgabaGaemOAaOgaaaaa@30A4@ denotes the *j*-th effector which can bind to the *i*-th binding domain *i*. We introduce labels for the status of the different binding sites such as 0 for an unoccupied binding domain and Eij
 MathType@MTEF@5@5@+=feaafiart1ev1aaatCvAUfKttLearuWrP9MDH5MBPbIqV92AaeXatLxBI9gBaebbnrfifHhDYfgasaacH8akY=wiFfYdH8Gipec8Eeeu0xXdbba9frFj0=OqFfea0dXdd9vqai=hGuQ8kuc9pgc9s8qqaq=dirpe0xb9q8qiLsFr0=vr0=vr0dc8meaabaqaciaacaGaaeqabaqabeGadaaakeaacqWGfbqrdaqhaaWcbaGaemyAaKgabaGaemOAaOgaaaaa@30A4@ for the domain *i *occupied with the *j*-th effector. Alternatively, the different states of a domain can also be enumerated. For instance, the two representations *R*[E14
 MathType@MTEF@5@5@+=feaafiart1ev1aaatCvAUfKttLearuWrP9MDH5MBPbIqV92AaeXatLxBI9gBaebbnrfifHhDYfgasaacH8akY=wiFfYdH8Gipec8Eeeu0xXdbba9frFj0=OqFfea0dXdd9vqai=hGuQ8kuc9pgc9s8qqaq=dirpe0xb9q8qiLsFr0=vr0=vr0dc8meaabaqaciaacaGaaeqabaqabeGadaaakeaacqWGfbqrdaqhaaWcbaGaeGymaedabaGaeGinaqdaaaaa@2FD2@, E21
 MathType@MTEF@5@5@+=feaafiart1ev1aaatCvAUfKttLearuWrP9MDH5MBPbIqV92AaeXatLxBI9gBaebbnrfifHhDYfgasaacH8akY=wiFfYdH8Gipec8Eeeu0xXdbba9frFj0=OqFfea0dXdd9vqai=hGuQ8kuc9pgc9s8qqaq=dirpe0xb9q8qiLsFr0=vr0=vr0dc8meaabaqaciaacaGaaeqabaqabeGadaaakeaacqWGfbqrdaqhaaWcbaGaeGOmaidabaGaeGymaedaaaaa@2FCE@, 0, E42
 MathType@MTEF@5@5@+=feaafiart1ev1aaatCvAUfKttLearuWrP9MDH5MBPbIqV92AaeXatLxBI9gBaebbnrfifHhDYfgasaacH8akY=wiFfYdH8Gipec8Eeeu0xXdbba9frFj0=OqFfea0dXdd9vqai=hGuQ8kuc9pgc9s8qqaq=dirpe0xb9q8qiLsFr0=vr0=vr0dc8meaabaqaciaacaGaaeqabaqabeGadaaakeaacqWGfbqrdaqhaaWcbaGaeGinaqdabaGaeGOmaidaaaaa@2FD4@] and *R*[4, 1, 0, 2] for a scaffold protein with four binding sites are equivalent. In addition, the representation *R*[*a*_1_, 0, 0, 0] denotes all scaffold proteins *R *with unoccupied binding domains 2 to 4 independent of the status of domain 1, etc.

All these definitions shall be clarified by considering a simple scaffold protein *R *with two domains 1 and 2 (*i *= 1, 2). Assuming it is known that binding domain 1 can bind three different effectors E11
 MathType@MTEF@5@5@+=feaafiart1ev1aaatCvAUfKttLearuWrP9MDH5MBPbIqV92AaeXatLxBI9gBaebbnrfifHhDYfgasaacH8akY=wiFfYdH8Gipec8Eeeu0xXdbba9frFj0=OqFfea0dXdd9vqai=hGuQ8kuc9pgc9s8qqaq=dirpe0xb9q8qiLsFr0=vr0=vr0dc8meaabaqaciaacaGaaeqabaqabeGadaaakeaacqWGfbqrdaqhaaWcbaGaeGymaedabaGaeGymaedaaaaa@2FCC@, E12
 MathType@MTEF@5@5@+=feaafiart1ev1aaatCvAUfKttLearuWrP9MDH5MBPbIqV92AaeXatLxBI9gBaebbnrfifHhDYfgasaacH8akY=wiFfYdH8Gipec8Eeeu0xXdbba9frFj0=OqFfea0dXdd9vqai=hGuQ8kuc9pgc9s8qqaq=dirpe0xb9q8qiLsFr0=vr0=vr0dc8meaabaqaciaacaGaaeqabaqabeGadaaakeaacqWGfbqrdaqhaaWcbaGaeGymaedabaGaeGOmaidaaaaa@2FCE@ and E13
 MathType@MTEF@5@5@+=feaafiart1ev1aaatCvAUfKttLearuWrP9MDH5MBPbIqV92AaeXatLxBI9gBaebbnrfifHhDYfgasaacH8akY=wiFfYdH8Gipec8Eeeu0xXdbba9frFj0=OqFfea0dXdd9vqai=hGuQ8kuc9pgc9s8qqaq=dirpe0xb9q8qiLsFr0=vr0=vr0dc8meaabaqaciaacaGaaeqabaqabeGadaaakeaacqWGfbqrdaqhaaWcbaGaeGymaedabaGaeG4mamdaaaaa@2FD0@ (*m*_1 _= 3 and *j *= 1, 2, 3), and that the second domain 2 can bind two different effectors E21
 MathType@MTEF@5@5@+=feaafiart1ev1aaatCvAUfKttLearuWrP9MDH5MBPbIqV92AaeXatLxBI9gBaebbnrfifHhDYfgasaacH8akY=wiFfYdH8Gipec8Eeeu0xXdbba9frFj0=OqFfea0dXdd9vqai=hGuQ8kuc9pgc9s8qqaq=dirpe0xb9q8qiLsFr0=vr0=vr0dc8meaabaqaciaacaGaaeqabaqabeGadaaakeaacqWGfbqrdaqhaaWcbaGaeGOmaidabaGaeGymaedaaaaa@2FCE@ and E22
 MathType@MTEF@5@5@+=feaafiart1ev1aaatCvAUfKttLearuWrP9MDH5MBPbIqV92AaeXatLxBI9gBaebbnrfifHhDYfgasaacH8akY=wiFfYdH8Gipec8Eeeu0xXdbba9frFj0=OqFfea0dXdd9vqai=hGuQ8kuc9pgc9s8qqaq=dirpe0xb9q8qiLsFr0=vr0=vr0dc8meaabaqaciaacaGaaeqabaqabeGadaaakeaacqWGfbqrdaqhaaWcbaGaeGOmaidabaGaeGOmaidaaaaa@2FD0@ (*m*_2 _= 2 and *j *= 1, 2). The number of feasible states of domain 1 is *m*_1 _+ 1 = 4, namely either unoccupied or occupied by one of the three effectors. According to these considerations the number of different states of domain 2 is *m*_2 _+ 1 = 3. The total number of complexes results from ∏(*m*_*i *_+ 1) = 4·3 = 12. In order to calculate the number of reactions that may occur in this example we first assume that domain 1 is unoccupied. Now three different effectors may bind to this domain, which corresponds to three different reactions. At the same time binding domain 2 can be unoccupied or occupied by one of the two effectors E2j
 MathType@MTEF@5@5@+=feaafiart1ev1aaatCvAUfKttLearuWrP9MDH5MBPbIqV92AaeXatLxBI9gBaebbnrfifHhDYfgasaacH8akY=wiFfYdH8Gipec8Eeeu0xXdbba9frFj0=OqFfea0dXdd9vqai=hGuQ8kuc9pgc9s8qqaq=dirpe0xb9q8qiLsFr0=vr0=vr0dc8meaabaqaciaacaGaaeqabaqabeGadaaakeaacqWGfbqrdaqhaaWcbaGaeGOmaidabaGaemOAaOgaaaaa@303B@. Hence, one has to consider 3·3 reactions describing the occupation of domain 1. Now we repeat these considerations for domain 2. This results in 2·4 reactions. The total number of reactions in our example is

∑i=1nmi∏k=1,k≠in(mk+1)=3⋅3+2⋅4=17.     (13)
 MathType@MTEF@5@5@+=feaafiart1ev1aaatCvAUfKttLearuWrP9MDH5MBPbIqV92AaeXatLxBI9gBaebbnrfifHhDYfgasaacH8akY=wiFfYdH8Gipec8Eeeu0xXdbba9frFj0=OqFfea0dXdd9vqai=hGuQ8kuc9pgc9s8qqaq=dirpe0xb9q8qiLsFr0=vr0=vr0dc8meaabaqaciaacaGaaeqabaqabeGadaaakeaadaaeWbqaaiabd2gaTnaaBaaaleaacqWGPbqAaeqaaaqaaiabdMgaPjabg2da9iabigdaXaqaaiabd6gaUbqdcqGHris5aOWaaebCaeaacqGGOaakcqWGTbqBdaWgaaWcbaGaem4AaSgabeaakiabgUcaRiabigdaXiabcMcaPiabg2da9iabiodaZiabgwSixlabiodaZaWcbaGaem4AaSMaeyypa0JaeGymaeJaeiilaWIaem4AaSMaeyiyIKRaemyAaKgabaGaemOBa4ganiabg+GivdGccqGHRaWkcqaIYaGmcqGHflY1cqaI0aancqGH9aqpcqaIXaqmcqaI3aWncqGGUaGlcaWLjaGaaCzcamaabmaabaGaeGymaeJaeG4mamdacaGLOaGaayzkaaaaaa@5C32@

### Reduction method

Our method can be divided into three essential steps. First, we start generating a complete mechanistic description of the considered scaffold or receptor like described in [1,14,15]. This step also includes the incorporation of qualitative information about domain interactions. Second step is the introduction of macro-states (levels of occupancy of each domain) using a state space transformation following a hierarchical pattern. The transformed system is reduced in a third step by eliminating all equations which do not influence the dynamics of the output variables y→
 MathType@MTEF@5@5@+=feaafiart1ev1aaatCvAUfKttLearuWrP9MDH5MBPbIqV92AaeXatLxBI9gBaebbnrfifHhDYfgasaacH8akY=wiFfYdH8Gipec8Eeeu0xXdbba9frFj0=OqFfea0dXdd9vqai=hGuQ8kuc9pgc9s8qqaq=dirpe0xb9q8qiLsFr0=vr0=vr0dc8meaabaqaciaacaGaaeqabaqabeGadaaakeaacuWG5bqEgaWcaaaa@2E39@. In the following we assume that one of the goals of signaling pathway modeling is to accurately describe the dynamics of the macro-states. Hence, we choose the levels of occupancy as output variables y→
 MathType@MTEF@5@5@+=feaafiart1ev1aaatCvAUfKttLearuWrP9MDH5MBPbIqV92AaeXatLxBI9gBaebbnrfifHhDYfgasaacH8akY=wiFfYdH8Gipec8Eeeu0xXdbba9frFj0=OqFfea0dXdd9vqai=hGuQ8kuc9pgc9s8qqaq=dirpe0xb9q8qiLsFr0=vr0=vr0dc8meaabaqaciaacaGaaeqabaqabeGadaaakeaacuWG5bqEgaWcaaaa@2E39@. This choice seems to be reasonable since these values correspond to experimentally verifiable quantities as discussed before. Additionally, similar examples can be found in literature where the same or very similar output quantities are defined [6,14,20]. Note that in principle one also may define other quantities of interest instead of the discussed levels of occupancy. Another choice of y→
 MathType@MTEF@5@5@+=feaafiart1ev1aaatCvAUfKttLearuWrP9MDH5MBPbIqV92AaeXatLxBI9gBaebbnrfifHhDYfgasaacH8akY=wiFfYdH8Gipec8Eeeu0xXdbba9frFj0=OqFfea0dXdd9vqai=hGuQ8kuc9pgc9s8qqaq=dirpe0xb9q8qiLsFr0=vr0=vr0dc8meaabaqaciaacaGaaeqabaqabeGadaaakeaacuWG5bqEgaWcaaaa@2E39@ would not affect the applicability of our method. However, it would modify the number of ODEs to be considered.

**Step 1: **The reaction networks considered are described by a system of ordinary differential equations (ODEs). All feasible reactions *A *+ *B *⇋ *C *are translated into reaction rates *r *= *k*_on_*c*_*A*_*c*_*B *_- *k*_off_*c*_*C *_using the law of mass action. Here, *k*_on _and *k*_off _denote the association and dissociation constants, while *c*_*i *_refers to the concentrations of the components. The ODEs for all feasible micro-states have the form

dcidt=∑rproduction−∑rcomsumption.     (4)
 MathType@MTEF@5@5@+=feaafiart1ev1aaatCvAUfKttLearuWrP9MDH5MBPbIqV92AaeXatLxBI9gBaebbnrfifHhDYfgasaacH8akY=wiFfYdH8Gipec8Eeeu0xXdbba9frFj0=OqFfea0dXdd9vqai=hGuQ8kuc9pgc9s8qqaq=dirpe0xb9q8qiLsFr0=vr0=vr0dc8meaabaqaciaacaGaaeqabaqabeGadaaakeaadaWcaaqaaiabdsgaKjabdogaJnaaBaaaleaacqWGPbqAaeqaaaGcbaGaemizaqMaemiDaqhaaiabg2da9maaqaeabaGaemOCai3aaSbaaSqaaiabbchaWjabbkhaYjabb+gaVjabbsgaKjabbwha1jabbogaJjabbsha0jabbMgaPjabb+gaVjabb6gaUbqabaaabeqab0GaeyyeIuoakiabgkHiTmaaqaeabaGaemOCai3aaSbaaSqaaiabbogaJjabb+gaVjabb2gaTjabbohaZjabbwha1jabb2gaTjabbchaWjabbsha0jabbMgaPjabb+gaVjabb6gaUbqabaaabeqab0GaeyyeIuoakiabc6caUiaaxMaacaWLjaWaaeWaaeaacqaI0aanaiaawIcacaGLPaaaaaa@5EBE@

In vector notation, Equation 4 can be written as x→˙=f→(x→,u→)
 MathType@MTEF@5@5@+=feaafiart1ev1aaatCvAUfKttLearuWrP9MDH5MBPbIqV92AaeXatLxBI9gBaebbnrfifHhDYfgasaacH8akY=wiFfYdH8Gipec8Eeeu0xXdbba9frFj0=OqFfea0dXdd9vqai=hGuQ8kuc9pgc9s8qqaq=dirpe0xb9q8qiLsFr0=vr0=vr0dc8meaabaqaciaacaGaaeqabaqabeGadaaakeaacuWG4baEgaWcgaGaaiabg2da9iqbdAgaMzaalaGaeiikaGIafmiEaGNbaSaacqGGSaalcuWG1bqDgaWcaiabcMcaPaaa@364E@, where x→
 MathType@MTEF@5@5@+=feaafiart1ev1aaatCvAUfKttLearuWrP9MDH5MBPbIqV92AaeXatLxBI9gBaebbnrfifHhDYfgasaacH8akY=wiFfYdH8Gipec8Eeeu0xXdbba9frFj0=OqFfea0dXdd9vqai=hGuQ8kuc9pgc9s8qqaq=dirpe0xb9q8qiLsFr0=vr0=vr0dc8meaabaqaciaacaGaaeqabaqabeGadaaakeaacuWG4baEgaWcaaaa@2E37@ denotes the vector of all considered micro-states and u→
 MathType@MTEF@5@5@+=feaafiart1ev1aaatCvAUfKttLearuWrP9MDH5MBPbIqV92AaeXatLxBI9gBaebbnrfifHhDYfgasaacH8akY=wiFfYdH8Gipec8Eeeu0xXdbba9frFj0=OqFfea0dXdd9vqai=hGuQ8kuc9pgc9s8qqaq=dirpe0xb9q8qiLsFr0=vr0=vr0dc8meaabaqaciaacaGaaeqabaqabeGadaaakeaacuWG1bqDgaWcaaaa@2E31@ denotes all possible input signals (e.g. extra-cellular ligand concentration in the case of a receptor at the cell membrane). The macro-states/output variables we are interested in will be denoted as y→=h→(x→)
 MathType@MTEF@5@5@+=feaafiart1ev1aaatCvAUfKttLearuWrP9MDH5MBPbIqV92AaeXatLxBI9gBaebbnrfifHhDYfgasaacH8akY=wiFfYdH8Gipec8Eeeu0xXdbba9frFj0=OqFfea0dXdd9vqai=hGuQ8kuc9pgc9s8qqaq=dirpe0xb9q8qiLsFr0=vr0=vr0dc8meaabaqaciaacaGaaeqabaqabeGadaaakeaacuWG5bqEgaWcaiabg2da9iqbdIgaOzaalaGaeiikaGIafmiEaGNbaSaacqGGPaqkaaa@33E7@, which can be calculated using x→
 MathType@MTEF@5@5@+=feaafiart1ev1aaatCvAUfKttLearuWrP9MDH5MBPbIqV92AaeXatLxBI9gBaebbnrfifHhDYfgasaacH8akY=wiFfYdH8Gipec8Eeeu0xXdbba9frFj0=OqFfea0dXdd9vqai=hGuQ8kuc9pgc9s8qqaq=dirpe0xb9q8qiLsFr0=vr0=vr0dc8meaabaqaciaacaGaaeqabaqabeGadaaakeaacuWG4baEgaWcaaaa@2E37@. Incorporating qualitative information about domain interactions helps to reduce the number of relevant parameters. For example, if it is known that two binding sites A and B are independent, the on- and off-rate constants do not change upon effector binding to the other domains. Note, that this is not a simplification, but merely a systematic incorporation of real physico-chemical constraints.

**Step 2: **In order to introduce new coordinates z→
 MathType@MTEF@5@5@+=feaafiart1ev1aaatCvAUfKttLearuWrP9MDH5MBPbIqV92AaeXatLxBI9gBaebbnrfifHhDYfgasaacH8akY=wiFfYdH8Gipec8Eeeu0xXdbba9frFj0=OqFfea0dXdd9vqai=hGuQ8kuc9pgc9s8qqaq=dirpe0xb9q8qiLsFr0=vr0=vr0dc8meaabaqaciaacaGaaeqabaqabeGadaaakeaacuWG6bGEgaWcaaaa@2E3B@ including the macro-states, we perform a linear transformation z→=Tx→
 MathType@MTEF@5@5@+=feaafiart1ev1aaatCvAUfKttLearuWrP9MDH5MBPbIqV92AaeXatLxBI9gBaebbnrfifHhDYfgasaacH8akY=wiFfYdH8Gipec8Eeeu0xXdbba9frFj0=OqFfea0dXdd9vqai=hGuQ8kuc9pgc9s8qqaq=dirpe0xb9q8qiLsFr0=vr0=vr0dc8meaabaqaciaacaGaaeqabaqabeGadaaakeaacuWG6bGEgaWcaiabg2da9iabdsfaujqbdIha4zaalaaaaa@31FD@, where *T *is a quadratic, non-singular matrix. The transformed model equations are

z→˙=Tx→˙=Tf→(x→,u→)|x→=T−1z→.     (5)
 MathType@MTEF@5@5@+=feaafiart1ev1aaatCvAUfKttLearuWrP9MDH5MBPbIqV92AaeXatLxBI9gBaebbnrfifHhDYfgasaacH8akY=wiFfYdH8Gipec8Eeeu0xXdbba9frFj0=OqFfea0dXdd9vqai=hGuQ8kuc9pgc9s8qqaq=dirpe0xb9q8qiLsFr0=vr0=vr0dc8meaabaqaciaacaGaaeqabaqabeGadaaakeaacuWG6bGEgaWcgaGaaiabg2da9iabdsfaujqbdIha4zaalyaacaGaeyypa0JaemivaqLafmOzayMbaSaacqGGOaakcuWG4baEgaWcaiabcYcaSiqbdwha1zaalaGaeiykaKYaaqqaaeaafaqabeGabaaabaaabaGafmiEaGNbaSaacqGH9aqpcqWGubavdaahaaWcbeqaaiabgkHiTiabigdaXaaakiqbdQha6zaalaaaaaGaay5bSdGaeiOla4IaaCzcaiaaxMaadaqadaqaaiabiwda1aGaayjkaiaawMcaaaaa@48FC@

In the following, we will introduce this transformation for a number of simple cases to exemplify our method and discuss the general pattern of this transformation. A strict mathematical and general formulation is also provided.

**Figure 1:**
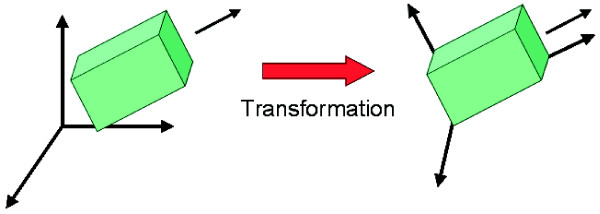
**State space transformation**. The idea of our method can be described more easily by considering a mechanical example: In order to model the movement of a mass in space one has to choose a certain coordinate system. However, if this coordinate system is not adjusted to the problem (like shown on the left site) the model equations will be more complicate than in a transformed coordinate system.

**Step 3: **Figure 1 shows that a state space transformation can simplify a model. In many relevant cases the transformed model equations of the considered scaffold proteins can be divided into two modules as shown in the following Equations

z→=[z→˙1z→˙2]=[g→1(z→1,u→)g→2(z→1,z→2,u→)],     (6)
 MathType@MTEF@5@5@+=feaafiart1ev1aaatCvAUfKttLearuWrP9MDH5MBPbIqV92AaeXatLxBI9gBaebbnrfifHhDYfgasaacH8akY=wiFfYdH8Gipec8Eeeu0xXdbba9frFj0=OqFfea0dXdd9vqai=hGuQ8kuc9pgc9s8qqaq=dirpe0xb9q8qiLsFr0=vr0=vr0dc8meaabaqaciaacaGaaeqabaqabeGadaaakeaacuWG6bGEgaWcaiabg2da9maadmaabaqbaeqabiqaaaqaaiqbdQha6zaalyaacaWaaSbaaSqaaiabigdaXaqabaaakeaacuWG6bGEgaWcgaGaamaaBaaaleaacqaIYaGmaeqaaaaaaOGaay5waiaaw2faaiabg2da9maadmaabaqbaeqabiqaaaqaaiqbdEgaNzaalaWaaSbaaSqaaiabigdaXaqabaGccqGGOaakcuWG6bGEgaWcamaaBaaaleaacqaIXaqmaeqaaOGaeiilaWIafmyDauNbaSaacqGGPaqkaeaacuWGNbWzgaWcamaaBaaaleaacqaIYaGmaeqaaOGaeiikaGIafmOEaONbaSaadaWgaaWcbaGaeGymaedabeaakiabcYcaSiqbdQha6zaalaWaaSbaaSqaaiabikdaYaqabaGccqGGSaalcuWG1bqDgaWcaiabcMcaPaaaaiaawUfacaGLDbaacqGGSaalcaWLjaGaaCzcamaabmaabaGaeGOnaydacaGLOaGaayzkaaaaaa@54B7@

y→=h→(z→1).     (7)
 MathType@MTEF@5@5@+=feaafiart1ev1aaatCvAUfKttLearuWrP9MDH5MBPbIqV92AaeXatLxBI9gBaebbnrfifHhDYfgasaacH8akY=wiFfYdH8Gipec8Eeeu0xXdbba9frFj0=OqFfea0dXdd9vqai=hGuQ8kuc9pgc9s8qqaq=dirpe0xb9q8qiLsFr0=vr0=vr0dc8meaabaqaciaacaGaaeqabaqabeGadaaakeaacuWG5bqEgaWcaiabg2da9iqbdIgaOzaalaGaeiikaGIafmOEaONbaSaadaWgaaWcbaGaeGymaedabeaakiabcMcaPiabc6caUiaaxMaacaWLjaWaaeWaaeaacqaI3aWnaiaawIcacaGLPaaaaaa@39BE@

Since *T *is a quadratic and non-singular (i.e. invertible) matrix these transformed equations still contain exactly the same information as the complete mechanistic model. Equation 6 shows that the macro-states (which are a subset of z→1
 MathType@MTEF@5@5@+=feaafiart1ev1aaatCvAUfKttLearuWrP9MDH5MBPbIqV92AaeXatLxBI9gBaebbnrfifHhDYfgasaacH8akY=wiFfYdH8Gipec8Eeeu0xXdbba9frFj0=OqFfea0dXdd9vqai=hGuQ8kuc9pgc9s8qqaq=dirpe0xb9q8qiLsFr0=vr0=vr0dc8meaabaqaciaacaGaaeqabaqabeGadaaakeaacuWG6bGEgaWcamaaBaaaleaacqaIXaqmaeqaaaaa@2F57@ and correspond to the output variables) are not influenced by the states z→2
 MathType@MTEF@5@5@+=feaafiart1ev1aaatCvAUfKttLearuWrP9MDH5MBPbIqV92AaeXatLxBI9gBaebbnrfifHhDYfgasaacH8akY=wiFfYdH8Gipec8Eeeu0xXdbba9frFj0=OqFfea0dXdd9vqai=hGuQ8kuc9pgc9s8qqaq=dirpe0xb9q8qiLsFr0=vr0=vr0dc8meaabaqaciaacaGaaeqabaqabeGadaaakeaacuWG6bGEgaWcamaaBaaaleaacqaIYaGmaeqaaaaa@2F59@. This implies that a reduced model only has to account for the ODEs z→˙1=g→1(z→1,u→)
 MathType@MTEF@5@5@+=feaafiart1ev1aaatCvAUfKttLearuWrP9MDH5MBPbIqV92AaeXatLxBI9gBaebbnrfifHhDYfgasaacH8akY=wiFfYdH8Gipec8Eeeu0xXdbba9frFj0=OqFfea0dXdd9vqai=hGuQ8kuc9pgc9s8qqaq=dirpe0xb9q8qiLsFr0=vr0=vr0dc8meaabaqaciaacaGaaeqabaqabeGadaaakeaacuWG6bGEgaWcgaGaamaaBaaaleaacqaIXaqmaeqaaOGaeyypa0Jafm4zaCMbaSaadaWgaaWcbaGaeGymaedabeaakiabcIcaOiqbdQha6zaalaWaaSbaaSqaaiabigdaXaqabaGccqGGSaalcuWG1bqDgaWcaiabcMcaPaaa@39CA@, and that this reduced model provides exactly the same output dynamics as the complete mechanistic model. However, note that by eliminating the equations for z→2
 MathType@MTEF@5@5@+=feaafiart1ev1aaatCvAUfKttLearuWrP9MDH5MBPbIqV92AaeXatLxBI9gBaebbnrfifHhDYfgasaacH8akY=wiFfYdH8Gipec8Eeeu0xXdbba9frFj0=OqFfea0dXdd9vqai=hGuQ8kuc9pgc9s8qqaq=dirpe0xb9q8qiLsFr0=vr0=vr0dc8meaabaqaciaacaGaaeqabaqabeGadaaakeaacuWG6bGEgaWcamaaBaaaleaacqaIYaGmaeqaaaaa@2F59@ one looses the possibility of using the inverse mapping *T*^-1 ^in order to retrieve the original variables x→
 MathType@MTEF@5@5@+=feaafiart1ev1aaatCvAUfKttLearuWrP9MDH5MBPbIqV92AaeXatLxBI9gBaebbnrfifHhDYfgasaacH8akY=wiFfYdH8Gipec8Eeeu0xXdbba9frFj0=OqFfea0dXdd9vqai=hGuQ8kuc9pgc9s8qqaq=dirpe0xb9q8qiLsFr0=vr0=vr0dc8meaabaqaciaacaGaaeqabaqabeGadaaakeaacuWG4baEgaWcaaaa@2E37@.

### Generic example with three binding domains

We will start with a scaffold protein with three binding domains. In our example, each domain can bind one distinct effector molecule. Hence, the scaffold protein can exist in eight different micro-states (*R*[0, 0, 0], *R*[1, 0, 0], *R*[0, 1, 0], *R*[0, 0, 1], *R*[1, 1, 0], *R *[1, 0, 1], *R*[0, 1, 1], *R*[1,1,1]). Even in this simple example 12 elementary reactions have to be considered (see Table 1).

#### Functionality of a scaffold protein

**Figure 2:**
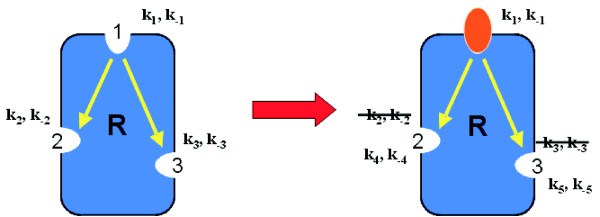
**Domain interactions**. We assume that binding domain one controls the other two domains like indicated by the arrows. From this assumption the kinetic parameters for the model follow immediately. As soon as binding domain one is occupied, the affinities of the docking sites two and three will change. Since binding domain one is independent of the other binding sites, the on- and off-rate constants of this domain are also independent.

Using the law of mass action and the reactions defined in Table 1, the model equations can be derived using Equation 4. However, besides the model structure the kinetic parameters play a crucial role in determining the system dynamics. A scaffold protein can perform a number of different functionalities, which can be characterized in terms of domain-interactions. One can think of a variety of cases such as non-interacting binding sites or the existence of a controlling domain influencing the others. A scaffold protein with 3 binding domains may provide 13 different functionalities or interaction motifs [21]. Qualitative knowledge about domain interactions have to be included in the modelling step. By defining the occuring domain interactions the number of relevant parameters follows immediately as exemplified in Figure 2. The assumption that, e.g., all three domains are completely independent implies that the kinetic on- and off-rate constants of one domain do not change upon effector binding at another domain. In the following, we want to show that for a number of interesting functionalities of scaffold proteins, we are able to find considerably reduced models using our method. Additionally, our method is also applicable to all other kind of functionalities which shall be exemplified considering a scaffold protein with four docking sites and a more complicated structure of occuring domain interactions. More detailed information about how the kinetic parameters have to be adjusted to realize a certain functionality can be found in the supplementary material (simulation files provided).

#### Hierarchical transformation

The second step after having formulated a complete mechanistic model is to perform a state space transformation. This transformation introduces new states, including the levels of occupancy of each domain. Choosing a globally invertible and smooth transformation assures that the system's dynamics is preserved and, as long as none of the new equations is eliminated, the original micro-states can be retrieved from the new ones at any time [22].

We choose a hierarchical transformation matrix, consisting of different tiers (Table 2). Each tier represents a certain level of detail. First, we define the 0th tier of our transformation matrix, which includes only one state representing the total concentrations of the scaffold protein (Equation 52a). Since there is no production nor degradation of *R *in our example, *z*_0 _will be constant. However, in the general case (including production and degradation) this is also an important dynamic and macroscopic quantity of interest. The 1st tier of our matrix corresponds to the discussed levels of occupancy of each binding domain (Equations 52b to 52d in our example). All states belonging to both of these tiers are called macro-states. The 2nd tier describes the levels of occupancy of all pairs of domains, corresponding to the concentration of scaffold proteins *R *with concurrently occupied binding domains {1,2}, {1,3} and {2,3} (Equations 52e to 52g in our example), which we call mesoscopic states. The 3rd and, in our example, last tier represents the levels of occupancy of all triples of domains corresponding to the micro-state *R*[1, 1, 1] (Equation 52h). The transformation matrix in the general case is structured completely similar. One starts with the overall concentration and the levels of occupancy, followed by all possible pairs, triples and higher tuples of concurrently occupied domains until one reaches the tier describing the micro-states. We define that all new states describing macroscopic properties as overall concentrations or levels of occupancy of distinct docking sites are called macro-states. Others describing pairs, triples or higher tuples of concurrently occupied binding sites are mesoscopic states and those specifying single multiprotein species correspond to the original micro-states. A generalized transformation applicable to scaffold proteins with *n *binding domains as well as a proof that any transformation matrix deduced with the described pattern is invertible is provided in the following. As mentioned above, the functionality of a scaffold protein or a receptor is uniquely determined by the choice of parameters. Hence, the transformation only depends on the number of binding domains and effectors, not on the functionality of the protein. This means that each scaffold protein with n docking sites has to be transformed in exactly the same way no matter which functionality it provides. However, the number of equations of the reduced model will of course depend on the functionality of the protein as will be shown in the following.

### Independent binding domains

First, a reduced model describing the example with three independent binding domains shall be introduced. The mechanistic model (Equation 4) is transformed as specified in Table 2. The transformed model equations have a very special structure (see Table 3). The total concentration of the scaffold protein *z*_0 _is constant. Additionally, the output variables (levels of occupancy) which are described by the states *z*_1 _to *z*_3 _in the model appear to be completely decoupled from all other ODEs. This finding implies that a model describing the levels of occupancy does not need to consider the whole set of ODEs. They are *accurately *described by Equations 53b to 53d. From the structure of the equations it can be also seen that all kinetic parameters of the model can only be identified if one has measurements for all three states.

### One site controls the others

**Figure 3:**
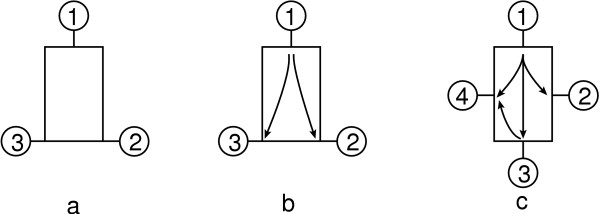
**Interaction motifs**. Generic examples of scaffold proteins with 3 or 4 docking sites. (a) A scaffold protein with 3 distinct docking sites which do not interact. (b) Another pattern of domain interactions for the same scaffold protein. Here binding domain 1 controls the other two domains. (c) A scaffold protein with 4 docking sites and a more complex pattern of domain interactions. Each pattern of domain interactions can be translated into special kinetic properties (like exemplified in Figure 2).

Now we assume that binding domain 1 controls the domains 2 and 3 (see Figure 3b). Again, this functionality corresponds to a special parameter combination, and the model is transformed using the same transformation as in the example above (see Table 2). In this case the states *z*_1 _to *z*_5 _are completely independent of states *z*_6 _and *z*_7 _(Table 4). As we are only interested in the levels of occupancy (states *z*_1 _to *z*_3_), the Equations for *z*_6 _and *z*_7 _can be excluded. Additionally, the Equations for *z*_1 _to *z*_5 _can be divided into three modules that are completely free of retroactive effects thus facilitating the analysis of the model as well as the application of parameter identification tools [23-25]. In comparison to the previous example two more equations are required in order to describe the dynamics of the macro-states. An explanation of this fact is provided by the following example. Consider the binding domains 1 and 2 of two equal scaffold molecules *R*. Only considering the macro-states ("domain 1 occupied" and "domain 2 occupied"), it is not possible to distinguish between the following two scenarios: [1] one molecule has both sites occupied and the other has none (*R*[1, 1,*a*_3_] and *R*[0, 0, *a*_3_]) and [2] one has the first site occupied, but not the second, and the other has the second, but not the first (*R*[1, 0, *a*_3_] and *R*[0,1, *a*_3_]). However, this information is essential if binding domain 2 is controlled by domain 1, since in the first case the affinity of *R*[0, 0, *a*_3_] to the effector E21
 MathType@MTEF@5@5@+=feaafiart1ev1aaatCvAUfKttLearuWrP9MDH5MBPbIqV92AaeXatLxBI9gBaebbnrfifHhDYfgasaacH8akY=wiFfYdH8Gipec8Eeeu0xXdbba9frFj0=OqFfea0dXdd9vqai=hGuQ8kuc9pgc9s8qqaq=dirpe0xb9q8qiLsFr0=vr0=vr0dc8meaabaqaciaacaGaaeqabaqabeGadaaakeaacqWGfbqrdaqhaaWcbaGaeGOmaidabaGaeGymaedaaaaa@2FCE@ has a different value from the second case *R*[1, 0, *a*_3_].

### Analysis and dynamic simulations

In order to illustrate the advantages of our method, the previous example will be discussed (see Figure 3b) in more detail, including dynamic simulations. Using the parameters listed in Table 5, we create three different models for this case: (i) a complete mechanistic description, (ii) an 'heuristically' reduced model, and (iii) an exactly reduced model according to the method described herein. Simulation results of the models are compared, and the advantages and disadvantages of the models are discussed.

**Model 1: **We create a complete mechanistic model accounting for all molecular species and all possible reactions (compare Table 1). Creating such a model one has to address the problem of combinatorial complexity. In this simple example the combinatorial variety of all feasible molecules includes only 11 different chemical species (8 complexes and 3 effectors). After incorporating conservation relations, the model only consists of 7 ODEs. However, most real problems would lead to models with tens of thousands or even millions of equations and the computational cost in these cases is extremely high. However, an advantage of such a detailed model is that it accurately represents the real reaction network. Now we want to compare the predictions of this complete mechanistic model with two different reduced models.

**Model 2: **We already mentioned that most heuristic models do not account for all molecular species. However, the equations of these models still describe the system at a microscopic level. The complete mechanistic network structure is substituted by a reduced structure focusing on a reduced number of species and a reduced number of reactions. As shown by Faeder et al. [12], which species and which reactions play an important role is highly dependent on the kinetic parameters, and it can hardly be found without the consideration of dynamic simulations of a complete mechanistic model. To illustrate the problems associated with this heuristic approach, we will show that even in our simple example a number of simplifications that may appear reasonable lead to a wrong model. The parameter values (see Table 5) show that if binding domain 1 is unoccupied the affinity of the other domains is extremely low. Hence, it may seem reasonable to neglect these reactions. Thus, the unoccupied receptor *R*[0, 0, 0] first has to bind the effector *E*_1_. Since the resulting affinity as well as the resulting on-rate of binding domain 2 is approximately several hundred-fold higher than the affinity or the on-rate of domain 3, we assume that the effector *E*_2 _in the majority of cases will bind before *E*_3_. Therefore, the reduced model only includes the following three reactions

*R*[0, 0, 0] + *E*_1 _⇋ *R*[1, 0, 0]     (8)

*R*[1, 0, 0] + *E*_2 _⇋ *R*[1, 1, 0]     (9)

*R*[1, 1, 0] + *E*_3 _⇋ *R*[1, 1, 1]     (10)

which are parametrized with the known kinetic constants. This case represents a commonly performed simplification. In [6], e.g. the two effectors GAP and Shc bind consecutively to the receptor, although the EGF receptor provides two distinct binding domains for these effectors similar to the scaffold in our example. In order to compare the predictions of this reduced model with the complete one we again consider the levels of occupancy of each domain. A comparison of the simulation results shows that the predictions of the reduced model, for these parameter values, are incorrect. Certainly, it would be possible to improve the predictions by fitting the parameter values using the curves provided by the correct model, but then the parameters would be phenomenological parameters and would not correspond to the kinetic parameters of the reactions. The general problem of models with such a linear chain of reactions is that the resulting levels of occupancy are always higher than one would expect considering the equilibrium constants of the reactions. The reason is quite simple. The equilibrium constant for reaction 8 determines the equilibrium between *R*[0, 0, 0], *E*_1 _and *R*[1, 0, 0]. However, in equilibrium only a small fraction of scaffold proteins which have bound *E*_1 _are in the state *R*[1, 0, 0]. Most of them have also bound *E*_2 _and *E*_3_, and the structure of Model 2 does not allow *E*_1 _to dissociate after *E*_2 _and *E*_3 _have bound to the scaffold protein.

**Figure 4:**
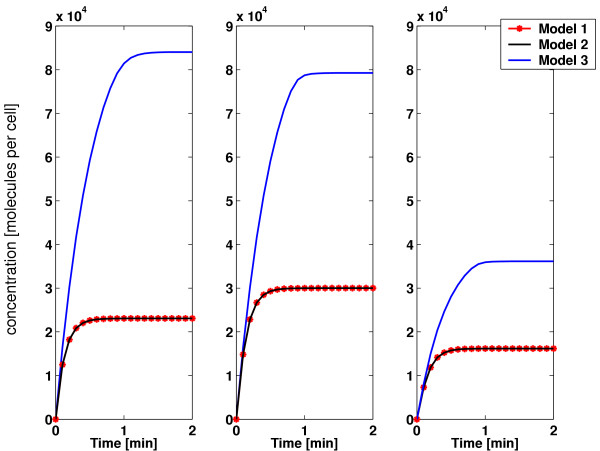
**Dynamic simulations**. Dynamic simulations of the example shown in Figure 3b using the parameter values presented in Table 5. Here we compare the levels of occupancy of the three protein domains. The left picture shows the level of occupancy of domain 1, the second picture shows the levels of occupancy of domain 2 and the right picture shows that of domain 3. The results show that the reduced model we obtained by applying our method (model 3) accurately describes the real time course represented by a complete mechanistic model (model 1). The other model which follows from a number of reasonable simplifications which can also be found in literature provides completely different results.

**Model 3: **At last we want to consider the reduced model, derived using our method (compare equations for *z*_1 _to *z*_5 _in Table 4). The dynamic simulations of the two models analyzed above show that both have disadvatages. The complete model is an accurate model describing all species and reactions, but the number of ODEs grows exponentially with the number of binding domains. While the second approach has the advantage that the number of equations is manageable, the error is not known. Our method combines the merits of both approaches: the number of equations is lower than in the complete model, but the predictions of both models are exactly the same (compare Figure 4). Additionally, our method gives rise to a very useful modular structure, which simplifies greatly the model analysis. In our example the model consists of three modules. The first module only includes the equation for *z*_1_, whereas the second consists of the two states *z*_2 _and *z*_4 _and the third of *z*_3 _and *z*_5_. The states of the second module do not influence the states of the third module and vice versa. The dynamics of *z*_1 _is neither influenced by the states of the second nor the third module, but *z*_1 _influences all the other states. Therefore, the model is not only modular but also hierarchically structured. This motivates a modular approach in order to analyze the model dynamics. First, one can analyze the dynamics of the first module. Afterwards the other two modules can also be analyzed separately. A similar approach is possible for the parameter identification since our transformation also leads to a modularization of the parameters. The only parameters of the first module are *k*_1 _and *k*_-1_. The parameters *k*_2_, *k*_-2_, *k*_3 _and *k*_-3 _can be found only in the second module, and the remaining parameters are present only in the third module. This allows one to identify the parameters step by step, which is a much simpler task than to identify all parameters at once. In addition, it also shows which parameters can be identified by certain measurements: for example, a measured time course of *z*_2 _(level of occupancy of domain 2) does not allow the identification of the parameters *k*_4_, *k*_-4_, *k*_5 _and *k*_-5_. The same also holds true for the complete model (model 1), but it is not intuitive to untangle this feature in the structure of the ODEs of the complete model. Because of all these advantages we think that the method proposed here offers a useful framework to handle multiprotein complex formation in signaling and regulation networks.

### Example with more than one controlling domain

In the previous chapter we analyzed scaffold proteins with completely independent binding domains or with only one controlling domain. However, our method can also be applied to a more general case. This will be exemplified by considering a scaffold protein with 4 binding domains, where each domain can be free or occupied by an effector. We assume that binding domain 1 controls domains 2, 3 and 4. Additionally, binding domain 3 also interacts with binding domain 4 (see Figure 3c). The number of feasible micro-states in this example is 2^4 ^= 16. Applying our method to this example (details can be found in the Appendix), one finds that 9 equations are sufficient to describe the complete dynamics of the macro-states. The states that are required are the levels of occupancy of all 4 binding domains, the pairs of concurrently occupied binding domains {1,2}, {1,3}, {1,4} and {3,4}, as well as the triple of concurrently occupied binding domains {1,3,4}. This result shows that the more binding domains interact, more ODEs are required in order to describe the dynamics of the macro-states. Indeed, if all binding domains interact with each other, one really has to consider the whole combinatorial variety.

### Scaffold proteins with n binding sites

#### Generalized transformation matrix

Here we want to formalize the transformation matrix for any scaffold protein with *n *binding sites *i*. Additionally, we assume that a number of *m*_*i *_effectors compete for the binding domain *a*_*i*_. Hence, each binding domain *i *can exist in *m*_*i *_+ 1 different states. The transformation matrix is independent of the intramolecular domain interactions. The 0th tier of our transformation matrix consists of only one state describing the overall concentration of the scaffold protein. The new state *z*_0 _corresponds to the sum of all feasible micro-states

z0=∑a1=0m1…∑an=0mnR[a1,…,an].     (11)
 MathType@MTEF@5@5@+=feaafiart1ev1aaatCvAUfKttLearuWrP9MDH5MBPbIqV92AaeXatLxBI9gBaebbnrfifHhDYfgasaacH8akY=wiFfYdH8Gipec8Eeeu0xXdbba9frFj0=OqFfea0dXdd9vqai=hGuQ8kuc9pgc9s8qqaq=dirpe0xb9q8qiLsFr0=vr0=vr0dc8meaabaqaciaacaGaaeqabaqabeGadaaakeaacqWG6bGEdaWgaaWcbaGaeGimaadabeaakiabg2da9maaqahabaGaeSOjGS0aaabCaeaacqWGsbGucqGGBbWwcqWGHbqydaWgaaWcbaGaeGymaedabeaakiabcYcaSiablAciljabcYcaSiabdggaHnaaBaaaleaacqWGUbGBaeqaaOGaeiyxa0LaeiOla4caleaacqWGHbqydaWgaaadbaGaemOBa4gabeaaliabg2da9iabicdaWaqaaiabd2gaTnaaBaaameaacqWGUbGBaeqaaaqdcqGHris5aaWcbaGaemyyae2aaSbaaWqaaiabigdaXaqabaWccqGH9aqpcqaIWaamaeaacqWGTbqBdaWgaaadbaGaeGymaedabeaaa0GaeyyeIuoakiaaxMaacaWLjaWaaeWaaeaacqaIXaqmcqaIXaqmaiaawIcacaGLPaaaaaa@5632@

The levels of occupancy of each binding domain are described in the 1st tier. The status of the binding domain *i *is fixed at the status *k *(with *k *∊ {1, ..., *m*_*i*_} ) and one sums up all micro-states whose binding domain *i *is occupied by the effector Eik
 MathType@MTEF@5@5@+=feaafiart1ev1aaatCvAUfKttLearuWrP9MDH5MBPbIqV92AaeXatLxBI9gBaebbnrfifHhDYfgasaacH8akY=wiFfYdH8Gipec8Eeeu0xXdbba9frFj0=OqFfea0dXdd9vqai=hGuQ8kuc9pgc9s8qqaq=dirpe0xb9q8qiLsFr0=vr0=vr0dc8meaabaqaciaacaGaaeqabaqabeGadaaakeaacqWGfbqrdaqhaaWcbaGaemyAaKgabaGaem4AaSgaaaaa@30A6@. Mathematically, this tier can be described by

z1=∑a2=0m2…∑an=0mnR[1,a2,…,an]     (12)⋮zm1=∑a2=0m2…∑an=0mnR[m1,a2,…,an]     (13)
 MathType@MTEF@5@5@+=feaafiart1ev1aaatCvAUfKttLearuWrP9MDH5MBPbIqV92AaeXatLxBI9gBaebbnrfifHhDYfgasaacH8akY=wiFfYdH8Gipec8Eeeu0xXdbba9frFj0=OqFfea0dXdd9vqai=hGuQ8kuc9pgc9s8qqaq=dirpe0xb9q8qiLsFr0=vr0=vr0dc8meaabaqaciaacaGaaeqabaqabeGadaaakeaafaqabeWacaaabaGaemOEaO3aaSbaaSqaaiabigdaXaqabaGccqGH9aqpdaaeWbqaaiablAcilnaaqahabaGaemOuaiLaei4waSLaeGymaeJaeiilaWIaemyyae2aaSbaaSqaaiabikdaYaqabaGccqGGSaalcqWIMaYscqGGSaalcqWGHbqydaWgaaWcbaGaemOBa4gabeaakiabc2faDbWcbaGaemyyae2aaSbaaWqaaiabd6gaUbqabaWccqGH9aqpcqaIWaamaeaacqWGTbqBdaWgaaadbaGaemOBa4gabeaaa0GaeyyeIuoaaSqaaiabdggaHnaaBaaameaacqaIYaGmaeqaaSGaeyypa0JaeGimaadabaGaemyBa02aaSbaaWqaaiabikdaYaqabaaaniabggHiLdaakeaacaWLjaGaaCzcamaabmaabaGaeGymaeJaeGOmaidacaGLOaGaayzkaaaabaGaeSO7I0eabaaabaGaemOEaO3aaSbaaSqaaiabd2gaTnaaBaaameaacqaIXaqmaeqaaaWcbeaakiabg2da9maaqahabaGaeSOjGS0aaabCaeaacqWGsbGucqGGBbWwcqWGTbqBdaWgaaWcbaGaeGymaedabeaakiabcYcaSiabdggaHnaaBaaaleaacqaIYaGmaeqaaOGaeiilaWIaeSOjGSKaeiilaWIaemyyae2aaSbaaSqaaiabd6gaUbqabaGccqGGDbqxaSqaaiabdggaHnaaBaaameaacqWGUbGBaeqaaSGaeyypa0JaeGimaadabaGaemyBa02aaSbaaWqaaiabd6gaUbqabaaaniabggHiLdaaleaacqWGHbqydaWgaaadbaGaeGOmaidabeaaliabg2da9iabicdaWaqaaiabd2gaTnaaBaaameaacqaIYaGmaeqaaaqdcqGHris5aaGcbaGaaCzcaiaaxMaadaqadaqaaiabigdaXiabiodaZaGaayjkaiaawMcaaaaaaaa@86DB@

zm1+1=∑a1=0m1∑a3=0m3…∑an=0mnR[a1,1,…,an](14)⋮zξ1=∑a1=0m1…∑an−1=0mn−1R[a1,…,an−1,mn],     (15)
 MathType@MTEF@5@5@+=feaafiart1ev1aaatCvAUfKttLearuWrP9MDH5MBPbIqV92AaeXatLxBI9gBaebbnrfifHhDYfgasaacH8akY=wiFfYdH8Gipec8Eeeu0xXdbba9frFj0=OqFfea0dXdd9vqai=hGuQ8kuc9pgc9s8qqaq=dirpe0xb9q8qiLsFr0=vr0=vr0dc8meaabaqaciaacaGaaeqabaqabeGadaaakeaafaqaaeWacaaabaGaemOEaO3aaSbaaSqaaiabd2gaTnaaBaaameaacqaIXaqmaeqaaSGaey4kaSIaeGymaedabeaakiabg2da9maaqahabaWaaabCaeaacqWIMaYsaSqaaiabdggaHnaaBaaameaacqaIZaWmaeqaaSGaeyypa0JaeGimaadabaGaemyBa02aaSbaaWqaaiabiodaZaqabaaaniabggHiLdGcdaaeWbqaaiabdkfasjabcUfaBjabdggaHnaaBaaaleaacqaIXaqmaeqaaOGaeiilaWIaeGymaeJaeiilaWIaeSOjGSKaeiilaWIaemyyae2aaSbaaSqaaiabd6gaUbqabaGccqGGDbqxaSqaaiabdggaHnaaBaaameaacqWGUbGBaeqaaSGaeyypa0JaeGimaadabaGaemyBa02aaSbaaWqaaiabd6gaUbqabaaaniabggHiLdaaleaacqWGHbqydaWgaaadbaGaeGymaedabeaaliabg2da9iabicdaWaqaaiabd2gaTnaaBaaameaacqaIXaqmaeqaaaqdcqGHris5aaGcbaWaaeWaaeaacqaIXaqmcqaI0aanaiaawIcacaGLPaaaaeGabaaGkiaaxMaacqWIUlstaeaaaeaacqWG6bGEdaWgaaWcbaGaeqOVdG3aaSbaaWqaaiabigdaXaqabaaaleqaaOGaeyypa0ZaaabCaeaacqWIMaYsdaaeWbqaaiabdkfasjabcUfaBjabdggaHnaaBaaaleaacqaIXaqmaeqaaOGaeiilaWIaeSOjGSKaeiilaWIaemyyae2aaSbaaSqaaiabd6gaUjabgkHiTiabigdaXaqabaGccqGGSaalcqWGTbqBdaWgaaWcbaGaemOBa4gabeaakiabc2faDjabcYcaSaWcbaGaemyyae2aaSbaaWqaaiabd6gaUjabgkHiTiabigdaXaqabaWccqGH9aqpcqaIWaamaeaacqWGTbqBdaWgaaadbaGaemOBa4MaeyOeI0IaeGymaedabeaaa0GaeyyeIuoaaSqaaiabdggaHnaaBaaameaacqaIXaqmaeqaaSGaeyypa0JaeGimaadabaGaemyBa02aaSbaaWqaaiabigdaXaqabaaaniabggHiLdGccaWLjaGaaCzcaaqaamaabmaabaGaeGymaeJaeGynaudacaGLOaGaayzkaaaaaaaa@9AB5@

with ξ1=∑i=1nmi
 MathType@MTEF@5@5@+=feaafiart1ev1aaatCvAUfKttLearuWrP9MDH5MBPbIqV92AaeXatLxBI9gBaebbnrfifHhDYfgasaacH8akY=wiFfYdH8Gipec8Eeeu0xXdbba9frFj0=OqFfea0dXdd9vqai=hGuQ8kuc9pgc9s8qqaq=dirpe0xb9q8qiLsFr0=vr0=vr0dc8meaabaqaciaacaGaaeqabaqabeGadaaakeaacqaH+oaEdaWgaaWcbaGaeGymaedabeaakiabg2da9maaqadabaGaemyBa02aaSbaaSqaaiabdMgaPbqabaaabaGaemyAaKMaeyypa0JaeGymaedabaGaemOBa4ganiabggHiLdaaaa@3A32@. The 2nd tier, which represents all pairs of concurrently occupied binding sites, corresponds to

zξ1+1=∑a3=0m3…∑an=0mnR[1,1,a3,…,an]     (16)⋮zξ2=∑a1=0m1…∑aj1−1=0mj1−1∑aj1+1=0mj1+1…∑aj2−1=0mj2−1∑aj2+1=0mj2+1…∑an=0mnR[a1,…,aj1−1,k,aj1+1,…aj2−1,l,aj2+1,…an]⋮     (17)
 MathType@MTEF@5@5@+=feaafiart1ev1aaatCvAUfKttLearuWrP9MDH5MBPbIqV92AaeXatLxBI9gBaebbnrfifHhDYfgasaacH8akY=wiFfYdH8Gipec8Eeeu0xXdbba9frFj0=OqFfea0dXdd9vqai=hGuQ8kuc9pgc9s8qqaq=dirpe0xb9q8qiLsFr0=vr0=vr0dc8meaabaqaciaacaGaaeqabaqabeGadaaakeaafaqabeabcaaaaeaacqWG6bGEdaWgaaWcbaGaeqOVdG3aaSbaaWqaaiabigdaXaqabaWccqGHRaWkcqaIXaqmaeqaaOGaeyypa0ZaaabCaeaacqWIMaYsdaaeWbqaaiabdkfasjabcUfaBjabigdaXiabcYcaSiabigdaXiabcYcaSiabdggaHnaaBaaaleaacqaIZaWmaeqaaOGaeiilaWIaeSOjGSKaeiilaWIaemyyae2aaSbaaSqaaiabd6gaUbqabaGccqGGDbqxaSqaaiabdggaHnaaBaaameaacqWGUbGBaeqaaSGaeyypa0JaeGimaadabaGaemyBa02aaSbaaWqaaiabd6gaUbqabaaaniabggHiLdaaleaacqWGHbqydaWgaaadbaGaeG4mamdabeaaliabg2da9iabicdaWaqaaiabd2gaTnaaBaaameaacqaIZaWmaeqaaaqdcqGHris5aaGcbaGaaCzcaiaaxMaadaqadaqaaiabigdaXiabiAda2aGaayjkaiaawMcaaaqaaiabl6UinbqaaaqaaiabdQha6naaBaaaleaacqaH+oaEdaWgaaadbaGaeGOmaidabeaaaSqabaGccqGH9aqpdaaeWbqaaiablAcilnaaqahabaWaaabCaeaacqWIMaYsaSqaaiabdggaHnaaBaaameaacqWGQbGAdaWgaaqaaiabigdaXaqabaGaey4kaSIaeGymaedabeaaliabg2da9iabicdaWaqaaiabd2gaTnaaBaaameaacqWGQbGAdaWgaaqaaiabigdaXaqabaGaey4kaSIaeGymaedabeaaa0GaeyyeIuoakmaaqahabaWaaabCaeaacqWIMaYsdaaeWbqaaiabdkfasjabcUfaBjabdggaHnaaBaaaleaacqaIXaqmaeqaaOGaeiilaWIaeSOjGSKaeiilaWIaemyyae2aaSbaaSqaaiabdQgaQnaaBaaameaacqaIXaqmaeqaaSGaeyOeI0IaeGymaedabeaakiabcYcaSiabdUgaRjabcYcaSiabdggaHnaaBaaaleaacqWGQbGAdaWgaaadbaGaeGymaedabeaaliabgUcaRiabigdaXaqabaGccqGGSaalcqWIMaYscqWGHbqydaWgaaWcbaGaemOAaO2aaSbaaWqaaiabikdaYaqabaWccqGHsislcqaIXaqmaeqaaOGaeiilaWIaemiBaWMaeiilaWIaemyyae2aaSbaaSqaaiabdQgaQnaaBaaameaacqaIYaGmaeqaaSGaey4kaSIaeGymaedabeaakiabcYcaSiablAciljabdggaHnaaBaaaleaacqWGUbGBaeqaaOGaeiyxa0faleaacqWGHbqydaWgaaadbaGaemOBa4gabeaaliabg2da9iabicdaWaqaaiabd2gaTnaaBaaameaacqWGUbGBaeqaaaqdcqGHris5aaWcbaGaemyyae2aaSbaaWqaaiabdQgaQnaaBaaabaGaeGOmaidabeaacqGHRaWkcqaIXaqmaeqaaSGaeyypa0JaeGimaadabaGaemyBa02aaSbaaWqaaiabdQgaQnaaBaaabaGaeGOmaidabeaacqGHRaWkcqaIXaqmaeqaaaqdcqGHris5aaWcbaGaemyyae2aaSbaaWqaaiabdQgaQnaaBaaabaGaeGOmaidabeaacqGHsislcqaIXaqmaeqaaSGaeyypa0JaeGimaadabaGaemyBa02aaSbaaWqaaiabdQgaQnaaBaaabaGaeGOmaidabeaacqGHsislcqaIXaqmaeqaaaqdcqGHris5aaWcbaGaemyyae2aaSbaaWqaaiabdQgaQnaaBaaabaGaeGymaedabeaacqGHsislcqaIXaqmaeqaaSGaeyypa0JaeGimaadabaGaemyBa02aaSbaaWqaaiabdQgaQnaaBaaabaGaeGymaedabeaacqGHsislcqaIXaqmaeqaaaqdcqGHris5aaWcbaGaemyyae2aaSbaaWqaaiabigdaXaqabaWccqGH9aqpcqaIWaamaeaacqWGTbqBdaWgaaadbaGaeGymaedabeaaa0GaeyyeIuoaaOqaaaqaaiabl6UinbqaaiaaxMaacaWLjaWaaeWaaeaacqaIXaqmcqaI3aWnaiaawIcacaGLPaaaaaaaaa@EED6@

with ξ2=∑i1=1nmi1+∑i1j1−1∑i2=0j2mi1mi2+kl
 MathType@MTEF@5@5@+=feaafiart1ev1aaatCvAUfKttLearuWrP9MDH5MBPbIqV92AaeXatLxBI9gBaebbnrfifHhDYfgasaacH8akY=wiFfYdH8Gipec8Eeeu0xXdbba9frFj0=OqFfea0dXdd9vqai=hGuQ8kuc9pgc9s8qqaq=dirpe0xb9q8qiLsFr0=vr0=vr0dc8meaabaqaciaacaGaaeqabaqabeGadaaakeaacqaH+oaEdaWgaaWcbaGaeGOmaidabeaakiabg2da9maaqadabaGaemyBa02aaSbaaSqaaiabdMgaPnaaBaaameaacqaIXaqmaeqaaaWcbeaaaeaacqWGPbqAdaWgaaadbaGaeGymaedabeaaliabg2da9iabigdaXaqaaiabd6gaUbqdcqGHris5aOGaey4kaSYaaabmaeaadaaeWaqaaiabd2gaTnaaBaaaleaacqWGPbqAdaWgaaadbaGaeGymaedabeaaaSqabaaabaGaemyAaK2aaSbaaWqaaiabikdaYaqabaWccqGH9aqpcqaIWaamaeaacqWGQbGAdaWgaaadbaGaeGOmaidabeaaa0GaeyyeIuoakiabd2gaTnaaBaaaleaacqWGPbqAdaWgaaadbaGaeGOmaidabeaaaSqabaaabaGaemyAaK2aaSbaaWqaaiabigdaXaqabaaaleaacqWGQbGAdaWgaaadbaGaeGymaedabeaaliabgkHiTiabigdaXaqdcqGHris5aOGaey4kaSIaem4AaSMaemiBaWgaaa@5B14@.

The following tiers represent all possible tuples, and the last tier of the transformation matrix (the *n *+ 1-th tier) contains the micro-states with all binding sites being occupied and can be written as

zξ3=R[1,…,1]     (18)⋮zξ4=R[m1,m2,…,mn]     (19)
 MathType@MTEF@5@5@+=feaafiart1ev1aaatCvAUfKttLearuWrP9MDH5MBPbIqV92AaeXatLxBI9gBaebbnrfifHhDYfgasaacH8akY=wiFfYdH8Gipec8Eeeu0xXdbba9frFj0=OqFfea0dXdd9vqai=hGuQ8kuc9pgc9s8qqaq=dirpe0xb9q8qiLsFr0=vr0=vr0dc8meaabaqaciaacaGaaeqabaqabeGadaaakeaafaqabeWacaaabaGaemOEaO3aaSbaaSqaaiabe67a4naaBaaameaacqaIZaWmaeqaaaWcbeaakiabg2da9iabdkfasjabcUfaBjabigdaXiabcYcaSiablAciljabcYcaSiabigdaXiabc2faDbqaaiaaxMaacaWLjaWaaeWaaeaacqaIXaqmcqaI4aaoaiaawIcacaGLPaaaaeaacqWIUlstaeaaaeaacqWG6bGEdaWgaaWcbaGaeqOVdG3aaSbaaWqaaiabisda0aqabaaaleqaaOGaeyypa0JaemOuaiLaei4waSLaemyBa02aaSbaaSqaaiabigdaXaqabaGccqGGSaalcqWGTbqBdaWgaaWcbaGaeGOmaidabeaakiabcYcaSiablAciljabcYcaSiabd2gaTnaaBaaaleaacqWGUbGBaeqaaOGaeiyxa0fabaGaaCzcaiaaxMaadaqadaqaaiabigdaXiabiMda5aGaayjkaiaawMcaaaaaaaa@5B67@

with ξ3=∏i=1n(mi+1)−∏i=1nmi
MathType@MTEF@5@5@+=feaafiart1ev1aaatCvAUfKttLearuWrP9MDH5MBPbIqV92AaeXatLxBI9gBaebbnrfifHhDYfgasaacH8akY=wiFfYdH8Gipec8Eeeu0xXdbba9frFj0=OqFfea0dXdd9vqai=hGuQ8kuc9pgc9s8qqaq=dirpe0xb9q8qiLsFr0=vr0=vr0dc8meaabaqaciaacaGaaeqabaqabeGadaaakeaacqaH+oaEdaWgaaWcbaGaeG4mamdabeaakiabg2da9maaradabaGaeiikaGIaemyBa02aaSbaaSqaaiabdMgaPbqabaGccqGHRaWkcqaIXaqmcqGGPaqkcqGHsisldaqeWaqaaiabd2gaTnaaBaaaleaacqWGPbqAaeqaaaqaaiabdMgaPjabg2da9iabigdaXaqaaiabd6gaUbqdcqGHpis1aaWcbaGaemyAaKMaeyypa0JaeGymaedabaGaemOBa4ganiabg+Givdaaaa@4831@ and ξ4=∏i=1n(mi+1)−1
MathType@MTEF@5@5@+=feaafiart1ev1aaatCvAUfKttLearuWrP9MDH5MBPbIqV92AaeXatLxBI9gBaebbnrfifHhDYfgasaacH8akY=wiFfYdH8Gipec8Eeeu0xXdbba9frFj0=OqFfea0dXdd9vqai=hGuQ8kuc9pgc9s8qqaq=dirpe0xb9q8qiLsFr0=vr0=vr0dc8meaabaqaciaacaGaaeqabaqabeGadaaakeaaliabe67a4naaBaaameaacqaI0aanaeqaaSGaeyypa0ZaaebmaeaacqGGOaakcqWGTbqBdaWgaaadbaGaemyAaKgabeaaliabgUcaRiabigdaXiabcMcaPaadbaGaemyAaKMaeyypa0JaeGymaedabaGaemOBa4gaoiabg+GivdWccqGHsislcqaIXaqmaaa@3FB9@. Using this pattern to derive the transformation matrix one can handle each possible scaffold protein.

#### Independent binding domains

Now we want to consider a scaffold protein with *n *binding sites and we assume that all binding domains are independent like we already did for a scaffold protein with three domains (see above). The parameters of the reaction network have to be adjusted to the case of independent binding domains as discussed above. The transformation matrix in this case also follows the hierarchical pattern as discussed in the section above. It can be shown that the whole dynamical behavior can be described by ∑ *m*_*i *_equations (i.e., states) instead of ∏ (*m*_*i *_+ 1) equations. A proof that in this case the macro-states are always sufficient to describe the system can also be found in [16].

#### One site controls the others

We also want to generalize the case that binding domain 1 controls all other (*n *- 1) binding domains, in a manner such that the affinity of each binding domain *i *to its respective effectors Eik
 MathType@MTEF@5@5@+=feaafiart1ev1aaatCvAUfKttLearuWrP9MDH5MBPbIqV92AaeXatLxBI9gBaebbnrfifHhDYfgasaacH8akY=wiFfYdH8Gipec8Eeeu0xXdbba9frFj0=OqFfea0dXdd9vqai=hGuQ8kuc9pgc9s8qqaq=dirpe0xb9q8qiLsFr0=vr0=vr0dc8meaabaqaciaacaGaaeqabaqabeGadaaakeaacqWGfbqrdaqhaaWcbaGaemyAaKgabaGaem4AaSgaaaaa@30A6@ only depends on the status of the binding domain 1. As already mentioned, the transformation matrix remains the same as for a protein with *n *independent binding domains. However, since the kinetic parameters are different, in this case more equations are required to describe the dynamics of the macro-states. In addition to the macro-states one requires all states describing pairs of concurrently occupied binding domain 1 and *i *with *i *≠ 1 (i.e. all states describing allosterically interacting domains). The total number of equations in this case is 2 ∑ *m*_*i *_- *m*_1 _instead of ∏ (*m*_*i *_+ 1).

### Linker for activation of T cells (LAT)

LAT (Linker for Activation of T cells) is a scaffold molecule that plays a pivotal role in T cell signaling [26]. LAT has 9 conserved cytoplasmatic tyrosines, of which the four membrane-distal tyrosines (at residues 132/171/191/226 in human LAT) are essential and are phosphorylated upon ligand binding to the T cell receptor [26]. Different signaling molecules, such as PLC*γ*1, Grb2 and Gads can bind to the different residues. Grb2 recruits Sos, which in turn activates Ras, and subsequently the Raf/MEK/ERK MAP Kinase cascade. On the other hand, binding of PLC*γ*1 and Gads (bound to the adaptor SLP76 that additionally recruits Itk), allows the activation of PLC*γ*1, leading to the cleavage of phosphatidyl-inositol-4,5 bisphosphate (PIP_2_) and the generation of dyacilglycerol (DAG) and inositol trisphosphate IP_3_. DAG activates RasGRP, which in turn activates Ras, as well as PKC, while IP_3 _regulates Calcium signaling [27].

PLC*γ*1 binds at the Y132 tyrosine, Grb2 at Y171, Y191 and Y226, and Gads at Y171 and Y191 (see Figure 4) [26]. The number of different protein complexes occurring in this simple example is already 2·3·3·2 = 36, and the number of reactions that have to be considered is 86. In the following we will show how one can precisely describe the levels of occupancy without considering all 36 complexes. First, it is assumed that the binding domains do not interact with each other. In order to reduce the model, the system has to be transformed using the transformation pattern discussed above. The transformed model equations show that only six differential equations are sufficient to completely describe the dynamics of the quantities of interest, namely the levels of occupancy of Y132, Y171, Y191 and Y226 with PLC*γ*1, Grb2 and Gads. Additionally, the equation describing PLC*γ*1 binding is decoupled from the other equations, while these are interlinked because Grb2 can bind to Y171, Y191 and Y226 and compete with Gads for the binding domains Y171 and Y191.

Recent experimental data from LAT mutation studies indicate that the binding domains can influence one another, which contradicts the assumption of the complete independence [28]. Binding of Grb2 to Y226 appears to help the binding of Gads to LAT [28]. This effect can be readily incorporated into the model by changing the kinetic parameters for Gads binding if the binding site Y226 is occupied by Grb2. Transforming the model equations shows that now ten ODEs are required to exactly describe the system dynamics (see supplementary information). The four additional states that are required here describe the number of LAT molecules, with concurrently occupied residues Y226 and either Y171 or Y191, while Y171 and Y191 can be occupied either by Grb2 or Gads. Although the model includes 4 additional states compared to the previously discussed one, the model can still be notably reduced from over thirty equations to ten. Hence, our method is capable of reducing signal transduction models including scaffold proteins notably. The modular structure of the derived model equations also strongly facilitates the model analysis as well as parameter estimation [25].

## Discussion

We have presented an approach which allows one to create reduced models of multiprotein complex formation processes often occuring in signal transduction cascades, but also in regulation mechanisms (e.g. cell cycle regulation [29]). Since each model reduction results in the loss of information, it is essential to define quantities of interest (output variables y→
 MathType@MTEF@5@5@+=feaafiart1ev1aaatCvAUfKttLearuWrP9MDH5MBPbIqV92AaeXatLxBI9gBaebbnrfifHhDYfgasaacH8akY=wiFfYdH8Gipec8Eeeu0xXdbba9frFj0=OqFfea0dXdd9vqai=hGuQ8kuc9pgc9s8qqaq=dirpe0xb9q8qiLsFr0=vr0=vr0dc8meaabaqaciaacaGaaeqabaqabeGadaaakeaacuWG5bqEgaWcaaaa@2E39@) whose dynamics should be preserved. As discussed in the background section, a reasonable choice are macroscopic values, such as levels of occupancy or phosphorylation degrees. Besides the determination of the output variables, the incorporation of qualitative knowledge about domain interactions is a key element in our approach. Both the choice of output variables as well as the considered domain interactions determine the number of ODEs in the reduced model. Hence, it is clear that for some patterns of domain interactions a reduction of the model may not be possible (e.g. if each domain interacts with all other domains). However, our preliminary results indicate that in many of these cases good approximate solutions can be found (data not shown). Since each possible pattern of domain interactions can be realized in the modeling step, and the state space transformation which has to be performed is completely independent of this interaction pattern, our method is generally applicable to all kind of molecules offering several binding sites. The only limitation is the possibility that no exact model reduction is possible which, however, is a general mathematical limitation and not an insufficiency of the method. Since our approach is very systematic, independent of exact numerical values for kinetic parameters and can be easily implemented as a computer algorithm, our hope is that this method will help to standardize, improve and simplify modeling and the analysis of important signaling networks.

## Conclusion

We have shown that a complete mechanistic model of scaffold proteins or receptors as discussed in [1,14,15], which describes the system at a microscopical level, can be linked in a analytical manner with the macroscopic and reduced description introduced in [16]. Our method is based on a linear state space transformation with a hierarchical structure (substituting the original mechanistic description by macroscopic, mesoscopic and a special set of microscopic states). It is a systematic and powerful tool to derive reduced model structures to describe the dynamics of multiprotein complex formation accurately.

## Appendix

### Proof

In order to prove that the transformation matrix is invertible, we will first show that the transformation matrix is quadratic, which is a necessary condition. Second, we will show that this matrix can be written as a upper triangular matrix, which is a sufficient condition for invertibility.

For each protein domain/site *i*, we introduce a set {*a*_*i*_, 1, ..., *m*_*i*_}, where the numbers 1, ..., *m*_*i *_denote possible states of site *i *and the character *a*_*i*_replaces 0, which was used to designate the basal state of site *i*. Having *n *such sets for all *n *domains (*i *= 1, ..., *n*), we select one element from each set and denote the resulting combination by z˜k
 MathType@MTEF@5@5@+=feaafiart1ev1aaatCvAUfKttLearuWrP9MDH5MBPbIqV92AaeXatLxBI9gBaebbnrfifHhDYfgasaacH8akY=wiFfYdH8Gipec8Eeeu0xXdbba9frFj0=OqFfea0dXdd9vqai=hGuQ8kuc9pgc9s8qqaq=dirpe0xb9q8qiLsFr0=vr0=vr0dc8meaabaqaciaacaGaaeqabaqabeGadaaakeaacuWG6bGEgaacamaaBaaaleaacqWGRbWAaeqaaaaa@2FC3@. Thus, any z˜k
 MathType@MTEF@5@5@+=feaafiart1ev1aaatCvAUfKttLearuWrP9MDH5MBPbIqV92AaeXatLxBI9gBaebbnrfifHhDYfgasaacH8akY=wiFfYdH8Gipec8Eeeu0xXdbba9frFj0=OqFfea0dXdd9vqai=hGuQ8kuc9pgc9s8qqaq=dirpe0xb9q8qiLsFr0=vr0=vr0dc8meaabaqaciaacaGaaeqabaqabeGadaaakeaacuWG6bGEgaacamaaBaaaleaacqWGRbWAaeqaaaaa@2FC3@ is a set of *n *elements where each element corresponds to one of the *n *domains and is either a number or a character (*a*_*i*_). There are ∏i=1n(mi+1)
MathType@MTEF@5@5@+=feaafiart1ev1aaatCvAUfKttLearuWrP9MDH5MBPbIqV92AaeXatLxBI9gBaebbnrfifHhDYfgasaacH8akY=wiFfYdH8Gipec8Eeeu0xXdbba9frFj0=OqFfea0dXdd9vqai=hGuQ8kuc9pgc9s8qqaq=dirpe0xb9q8qiLsFr0=vr0=vr0dc8meaabaqaciaacaGaaeqabaqabeGadaaakeaadaqeWaqaaiabcIcaOiabd2gaTnaaBaaaleaacqWGPbqAaeqaaOGaey4kaSIaeGymaeJaeiykaKcaleaacqWGPbqAcqGH9aqpcqaIXaqmaeaacqWGUbGBa0Gaey4dIunaaaa@39CB@ different combinations z˜k
 MathType@MTEF@5@5@+=feaafiart1ev1aaatCvAUfKttLearuWrP9MDH5MBPbIqV92AaeXatLxBI9gBaebbnrfifHhDYfgasaacH8akY=wiFfYdH8Gipec8Eeeu0xXdbba9frFj0=OqFfea0dXdd9vqai=hGuQ8kuc9pgc9s8qqaq=dirpe0xb9q8qiLsFr0=vr0=vr0dc8meaabaqaciaacaGaaeqabaqabeGadaaakeaacuWG6bGEgaacamaaBaaaleaacqWGRbWAaeqaaaaa@2FC3@, exactly equaling the number of micro-states (columns in the transformation matrix T). The transformation of combination z˜k
 MathType@MTEF@5@5@+=feaafiart1ev1aaatCvAUfKttLearuWrP9MDH5MBPbIqV92AaeXatLxBI9gBaebbnrfifHhDYfgasaacH8akY=wiFfYdH8Gipec8Eeeu0xXdbba9frFj0=OqFfea0dXdd9vqai=hGuQ8kuc9pgc9s8qqaq=dirpe0xb9q8qiLsFr0=vr0=vr0dc8meaabaqaciaacaGaaeqabaqabeGadaaakeaacuWG6bGEgaacamaaBaaaleaacqWGRbWAaeqaaaaa@2FC3@ into the variable *z*_*k *_is straightforward: summing up all micro-states that (1) correspond to each character entry *a*_*i *_(from *i *= 0 to *m*_*i*_) and (2) have the states of the other domain given by the numbers in z˜k
 MathType@MTEF@5@5@+=feaafiart1ev1aaatCvAUfKttLearuWrP9MDH5MBPbIqV92AaeXatLxBI9gBaebbnrfifHhDYfgasaacH8akY=wiFfYdH8Gipec8Eeeu0xXdbba9frFj0=OqFfea0dXdd9vqai=hGuQ8kuc9pgc9s8qqaq=dirpe0xb9q8qiLsFr0=vr0=vr0dc8meaabaqaciaacaGaaeqabaqabeGadaaakeaacuWG6bGEgaacamaaBaaaleaacqWGRbWAaeqaaaaa@2FC3@, we obtain the variable z˜k
 MathType@MTEF@5@5@+=feaafiart1ev1aaatCvAUfKttLearuWrP9MDH5MBPbIqV92AaeXatLxBI9gBaebbnrfifHhDYfgasaacH8akY=wiFfYdH8Gipec8Eeeu0xXdbba9frFj0=OqFfea0dXdd9vqai=hGuQ8kuc9pgc9s8qqaq=dirpe0xb9q8qiLsFr0=vr0=vr0dc8meaabaqaciaacaGaaeqabaqabeGadaaakeaacuWG6bGEgaacamaaBaaaleaacqWGRbWAaeqaaaaa@2FC3@. We conclude that the number of rows in the matrix *T *(the variables *Z*_*k*_) equals the number of columns (the micro-states).

The next step is to proof that all columns of the transformation matrix are linearly independent by complete induction. If we look consecutively at all the new defined variables, starting with the last one in our listing above, one can show that each state is a sum of already defined states plus *one *additional new state. Hence, the transformation matrix is an upper triangular matrix, and an upper triangular matrix has linearly independent columns and is hence invertible. Considering the last tier of our transformation matrix, which corresponds to a number of different micro-states, it is obvious that in each column a new micro-state is introduced. The *i*-th tier is a sum of micro-states, which have *i *- 1 binding domains with a well-defined status. In contrast, the status of the remaining binding domains can vary. One possible state is that all *n *- *i *+ 1 undetermined binding domains are unoccupied. This state is introduced here the first time, since all previous tiers consist of micro-states with a higher number of binding domains with well-defined status (excluding the unoccupied status). Hence, the number of unoccupied binding domains in the previous tiers is lower and cannot contain the described micro-state.

### Example with more than one controlling domain

In this example we assume that the considered scaffold molecule has four binding domains (1, 2, 3 and 4), which can bind four distinct effectors *E*_1_, *E*_2_, *E*_3 _and *E*_4_. The domain-domain interactions are depicted in Figure 1c (Binding domain 1 controls all other domains and binding domain 3 additionally influences binding domain 4). A mechanistic model accounting for all possible reactions of this scaffold molecule *R *with the effectors consists of 16 ordinary differential equations and incorporates 32 reactions. We provide a Mathematica-File, including all reactions, the mechanistic model equations as well as the transformation and the resulting reduced model. As already denoted, the reduced model consist of 9 ordinary differential equation, namely

*ż*_0_* = *0     (20)

*ż*_1 _= *k*_1_(*z*_0 _- *z*_1_)*E*_1 _- *k*_-1_*z*_1 _    (21)

*ż*_2 _= *k*_2_(*z*_0 _- *z*_1 _- * z*_2 _+ *z*_5_)*E*_2 _- *k*_-2_(*z*_2 _- *z*_5_) + *k*_3_(*z*_1 _- *z*_5_)*E*_2 _- *k*_-3_*z*_5 _    (22)

*ż*_3 _= *k*_4_(*z*_0 _- *z*_1 _- *z*_3 _+ *z*_6_)*E*_3 _- *k*_-4 _(*z*_3 _- *z*_6_) + *k*_5_(*z*_1_- *z*_6_)*E*_3 _- *k*_-5_*z*_6 _    (23)

*ż*_4 _= *k*_6_(*z*_0 _- *z*_1 _+ *z*_10 _- *z*_13 _- *z*_3 _- *z*_4 _+ *z*_6 _+ *z*_7_)*E*_4 _- *k*_-6_(*z*_4 _- *z*_7 _- *z*_10 _+ *z*_13_)     (24)

    *k*_7_(*z*_1 _- *z*_6 _- *z*_7 _+ *z*_13_)*E*_4 _- * k*_-7_(*z*_7 _- * z*_13_) + *k*_8_(*z*_3 _- * z*_6 _- * z*_10 _+ *z*_13_)*E*_4 _

    - *k*_-8_(*z*_10 _- * z*_13_) + *k*_9_(*z*_6 _- * z*_13_)*E*_4 _- * k*_-9_*z*_13 _    (25)

*ż*_5 _= *k*_1_(*z*_2 _- * z*_5_)*E*_1 _- * k*_-1_*z*_5 _+ *k*_3_(*z*_1 _- * z*_5_) - *k*_-3_*z*_5 _    (26)

*ż*_6 _= *k*_1_(*z*_3 _- * z*_6_)*E*_1 _- * k-*_1_*z*_6 _+ *k*_5_(*z*_1 _- * z*_6_) - *k*_-5_*z*_6 _    (27)

*ż*_7 _= *k*_1_(*z*_4 _- *z*_7_)*E*_1 _- *k*_-1_*z*_7 _+ *k*_9_(*z*_6 _- *z*_13_)*E*_4 _- *k*_-9_*z*_13 _    (28)

    + *k*_7_(*z*_1 _- * z*_6 _- * z*_7 _+ *z*_13_)*E*_4 _- * k*_-7_(*z*_7 _- * z*_13_)

*ż*_10 _= *k*_4 _(*z*_4 _- *z*_7 _- *z*_10 _+ *z*_13_)*E*_3 _- *k*_-4_(*z*_10 _*- z*_13_) + *k*_5_(*z*_7 _- *z*_13_)*E*_3 _- *k*_-5_*z*_13 _    (29)

    + *k*_8_(*z*_3 _- * z*_6 _- * z*_10 _+ *z*_13_)*E*_4 _- * k*_-8_(*z*_10 _*- z*_13_) + *k*_9_(*z*_6 _- * z*_13_)*E*_4 _- k_-9_*z*_13_

*ż*_13 _= *k*_1_(*z*_10 _- * z*_13_)*E*_1 _- * k*_-1_*z*_13 _+ *k*_5_(*z*_7 _- * z*_13_) - *k*_-5_*z*_13 _    (30)

    + *k*_9_(*z*_6 _- *z*_13_)*E*_4 _- *k*_-9_*z*_13_.

In these differential equations *z*_0 _denotes the overall concentration of the scaffold protein. The states *z*_1 _to *z*_4 _represent the levels of occupancy of the four distinct binding domains. The remaining states describe the number of scaffold proteins with two or three concurrently occupied binding domains ( *z*_5 _corresponds to concurrently occupied binding domains {1,2}, *z*_6 _equals {1,3}, *z*_7 _{1,4}, *z*_10 _{3,4} and *z*_13 _{1,3,4}).

### Linker for activation of T cells (LAT)

Applying our method to the adaptor protein LAT (Linker for activation of T cells), we derived two different reduced models (dependent of the assumptions that are made). First, we assume that the binding residues Y132, Y171, Y191 and Y226 are completely independent (see Figure 2). Hence, the reduced model only includes six differential equations. The derivation of these equations as well as the reactions and the mechanistic model equations are provided as a Mathematica-File. The reduced model equations are

*ż*_0 _= 0     (31)

*ż*_1 _= *k*_1_(*z*_0 _- * z*_1_)*PLC *- *k*_-1_*z*_1 _    (32)

*ż*_2 _= *k*_2_(*z*_0 _- *z*_2 _- *z*_3_)*Grb*2- *k*_-2_*z*_2 _    (33)

*ż*_3 _= *k*_5_(*z*_0 _- * z*_2 _- * z*_3_)*Gads *- *k*_-5_*z*_3 _    (34)

*ż*_4 _= *k*_3_(*z*_0 _- *z*_4 _- *z*_5_)*Grb*2 - *k*_-3_*z*_4 _    (35)

*ż*_5 _= *k*_6_(*z*_0 _- *z*_4 _- *z*_5_)*Gads *- *k*_-6_*z*_5 _    (36)

*ż*_6 _= *k*_4_(*z*_0 _- *z*_6_)*Grb*2 - *k*_-4_*z*_6_.     (37)

The state *z*_0 _denotes the overall concentration of LAT molecules, which is assumed to be constant. The state *z*_1 _equals the level of occupancy of Y132 with PLC*γ*1. *z*_2 _and *z*_3 _describe the levels of occupancy of Y171 either with Grb2 or Gads, *z*_4 _and *z*_5 _are the equivalent values for the binding domain Y191, and *z*_6 _denotes the level of occupancy of Y226 with Grb2.

However, recent experimental results show that the binding domains of LAT influence each other. In a second example we assume that Y226 interacts with the two domains Y171 and Y191. The whole calculation can be found in the provided Mathematica-File. In this case the reduced model consists of ten ordinary differential equations, namely

*ż*_1 _= *k*_1_(*z*_0 _- *z*_1_)*PLC *- *k*_-1_*z*_1 _    (38)

*ż*_2 _= *k*_2_(*z*_0 _- *z*_2 _- *z*_3_)*Grb*2 - *k*_-2_*z*_2 _    (39)

*ż*_3 _= *k*_5_(*z*_0 _- * z*_2 _- * z*_3 _- * z*_6 _+ *z*_14 _+ *z*_17_)*Gads *- *k*_-5_(*z*_3 _- * z*_17_)     (40)

    + *k*_6_(*z*_6 _- *z*_14 _- *z*_17_)*Gads *- *k*_-6_z_17_

*ż*_4 _= *k*_3_(*z*_0 _- * z*_4 _- * z*_5_)*Grb*2 - *k*_-3_*z*_4 _    (41)

*ż*_5 _= *k*_7_(*z*_0 _- *z*_4 _- *z*_5 _- *z*_6 _+ *z*_18 _+ *z*_19_)*Gads *- *k*_-7_(*z*_5 _- * z*_19_)     (42)

    + *k*_8_(*z*_6 _- * z*_18 _- * z*_19_)*Gads *- *k*_-6_*z*_19_

*ż*_6 _= *k*_4_(*z*_0 _- *z*_6_)*Grb*2 - *k*_-4_*z*_6 _    (43)

*ż*_14 _= *k*_2_(*z*_6 _- *z*_14 _- *z*_17_)*Grb*2 - *k*_-2_*z*_14 _+ *k*_4_(*z*_2 _- *z*_14_)*Grb*2 - *k*_-4_z_14 _    (44)

*ż*_17 _= *k*_4_(*z*_3 _- *z*_17_)*Grb*2 - *k*_-4_*z*_17 _+ *k*_6_(*z*_6 _- *z*_14 _- *z*_17_)*Gads *- *k*_-6_*z*_17 _    (45)

*ż*_18 _*= k*_3_(*z*_6 _- * z*_18 _- * z*_19_)*Grb*2 - *k*_-3_*z*_18 _+ *k*_4 _(*z*_4 _- * z*_18_)*Grb*2 - *k*_-4_*z*_18 _    (46)

*ż*_19 _= *k*_4_(*z*_5 _- *z*_19_)*Grb*2 - *k*_-4_*z*_19 _+ *k*_8_(*z*_6 _- *z*_18 _- *z*_19_)*Gads *- *k*_-8_*z*_19_.     (47)

    (48)

Again the states *z*_0 _to *z*_6 _correspond to the same quantities as described above. The state *z*_14 _equals to all LAT molecules with Grb2 being concurrently bound to the residues Y171 and Y226, *z*_17 _describes the molecules with Gads being bound to Y171 and Grb2 bound to Y226. The states *z*_18 _and *z*_19 _are equivalent quantities describing the same binding motifs for Y191 and Y226.

## Authors' contributions

HC conceived the original mathematical idea. HC, JSR and BNK developed the systematic method.

Model building and numerical analysis were carried out by HC, JSR and TS. EDG initiated, supervised and coordinated the project. All authors wrote the manuscript and approved the final version.

## Supplementary Material

Additional File 1 Additional Files can be downloaded from . All Files are created with Mathematica Version 5.0. The five files provide all information about the discussed examples. • Generic example with three binding domains (no domain interactions). • Generic example with three binding domains (one controlling domain). • Dynamic simulations of three distinct models. • Generic example with four binding domains. • LAT example (Linker for activation of T-Cells) Click here for file

## Figures and Tables

**Figure 5:**
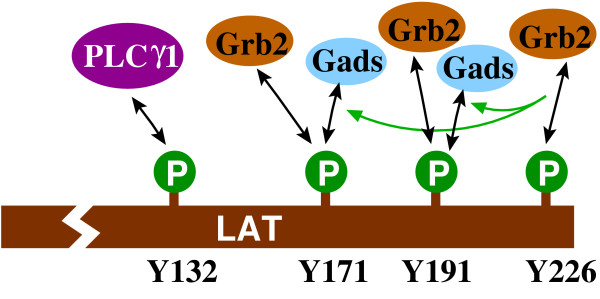
**Linker for activation of T-cells**. The four distal tyrosine rests on LAT and the binding possibilities, according to [26, 28].

**Table 1:** Reactions for scaffold with 3 binding sites. A complete mechanistic model of a scaffold protein with 3 binding domains (1,2,3), where each domain can bind one effector protein (*E*_1_, *E*_2_, *E*_3_), has to consider the following 12 reactions. The kinetic parameters for each reaction can be denoted with *k*_+*i *_for the association and *k*_-*i *_for the dissociation reaction

Binding of *E*_1_	Binding of *E*_2_	Binding of *E*_3_
*R*[0, 0, 0]+*E*_1 _⇋ *R*[1, 0, 0] (49a)	*R*[0, 0, 0]+*E*_2 _⇋ *R*[0, 1, 0] (50a)	*R*[0, 0, 0]+*E*_3 _⇋ *R*[0, 0, 1] (51a)
*R*[0, 1, 0]+*E*_1 _⇋ *R*[1, 1, 0] (49b)	*R*[0, 0, 1]+*E*_2 _⇋ *R*[0, 1, 1] (50b)	*R*[0, 1, 0]+*E*_3 _⇋ *R*[0, 1, 1] (51b)
*R*[0, 0, 1]+*E*_1 _⇋ *R*[1, 0, 1] (49c)	*R*[1, 0, 0]+*E*_2 _⇋ *R*[1, 1, 0] (50c)	*R*[1, 0, 0]+*E*_3 _⇋ *R*[1, 0, 1] (51c)
*R*[0, 1, 1]+*E*_1 _⇋ *R*[1, 1, 1] (49d)	*R*[1, 0, 1]+*E*_2 _⇋ *R*[1, 1, 1] (50d)	*R*[1, 1, 0]+*E*_3 _⇋ *R*[1, 1, 1] (51d)

**Table 2:** State Space Transformation for scaffold with three binding domains. Transformation for a scaffold protein with 3 binding domains. The transformation is hierarchically structured and introduces macroscopic quantities like the overall concentration of *R *and the levels of occupancy of each domain (*z*_0 _to *z*_3_), mesoscopic quantities describing pairs of concurrently occupied domains (*z*_4 _to *z*_6_) and microscopic quantities corresponding to individual multiprotein species (*z*_7_).

*z*_0 _= *R*[0, 0, 0] + *R*[1, 0, 0] + *R*[0, 1, 0] + *R*[0, 0, 1] + *R*[1, 1, 0] + *R*[1, 0, 1] + *R*[0, 1, 1] + *R*[1, 1, 1] (52a)
*z*_1 _= *R*[1, 0, 0] + *R*[1, 1, 0] + *R*[1, 0, 1] + *R*[1, 1, 1] (52b)
*z*_2 _= *R*[0, 1, 0] + *R*[1, 1, 0] + *R*[0, 1, 1] + *R*[1, 1, 1] (52c)
*z*_3 _= *R*[0, 0, 1] + *R*[1, 0, 1] + *R*[0, 1, 1] + *R*[1, 1, 1] (52d)
*z*_4 _= *R*[1, 1, 0] + *R*[1, 1, 1] (52e)
*z*_5 _= *R*[1, 0, 1] + *R*[1, 1, 1] (52f)
*z*_6 _= *R*[0, 1, 1] + *R*[1, 1, 1] (52g)
*z*_7 _= *R*[1, 1, 1] (52h)

**Table 3:** Transformed equations for independent domains. Transformed model equations for a scaffold protein with independent binding domains. The levels of occupancy (*z*_1 _to *z*_3_) do not depend on the states *z*_4 _to *z*_7_.

*ż*_0 _= 0 (53a)
*ż*_1 _= *k*_1 _(*z*_0 _- *z*_1_) *E*_1 _- *k*_-1_*z*_1 _ (53b)
*ż*_2 _= *k*_2 _(*z*_0 _- *z*_2_) *E*_2 _- *k*_-2_*z*_2 _ (53c)
*ż*_3 _= *k*_3 _(*z*_0 _- *z*_3_) *E*_3 _- *k*_-3_*z*_3 _ (53d)
*ż*_4 _= *k*_1 _(*z*_2 _- *z*_4_) *E*_1 _- *k*_-1_*z*_4 _+ *k*_2 _(*z*_1 _- *z*_4_) *E*_2 _- *k*_-2_*z*_4 _ (53e)
*ż*_5 _= *k*_1 _(*z*_3 _- *z*_5_) *E*_1 _- *k*_-1_*z*_5 _+ *k*_3 _(*z*_1 _- *z*_5_) *E*_3 _- *k*_-3_*z*_5 _ (53f)
*ż*_6 _= *k*_2 _(*z*_3 _- *z*_6_) *E*_2 _- *k*_-2_*z*_6 _+ *k*_3 _(*z*_2 _- *z*_6_) *E*_3 _- *k*_-3_*z*_6 _ (53g)
*ż*_7 _= *k*_1 _(*z*_6 _- *z*_7_) *E*_1 _- *k*_-1_*z*_7 _+ *k*_2 _(*z*_5 _- *z*_7_) *E*_2 _- *k*_-2_*z*_7 _+ *k*_3 _(*z*_4 _- *z*_7_) *E*_3 _- *k*_-3_*z*_7_. (53h)

**Table 4:** Transformed ODEs for scaffold with on controlling domain. Transformed model equations for a scaffold protein with one controlling domain. The levels of occupancy (*z*_1 _to *z*_3_) are only influenced by the states *z*_4 _and *z*_5 _but not by *z*_6 _and *z*_7_.

*ż*_0 _= 0 (54a)
*ż*_1 _= *k*_1 _(*z*_0 _- *z*_1_) *E*_1 _- *k*_-1_*z*_1 _ (54b)
*ż*_2 _= *k*_2 _(*z*_0 _- *z*_1 _- *z*_2 _+ *z*_4_) *E*_2 _- *k*_-2 _(*z*_2 _- *z*_4_) + *k*_3 _(*z*_1 _- *z*_4_) *E*_2 _- *k*_-3_*z*_4 _ (54c)
*ż*_3 _= *k*_4 _(*z*_0 _- *z*_1 _- *z*_3 _+ *z*_5_) *E*_3 _- *k*_-4 _(*z*_3 _- *z*_5_) + *k*_5 _(*z*_1 _- *z*_5_) *E*_3 _- *k*_-5_*z*_5 _ (54d)
*ż*_4 _= *k*_1_*E*_1 _(*z*_2 _- *z*_4_) - *k*_-1_*z*_4 _+ *k*_3_*E*_2 _(*z*_1 _- *z*_4_) - *k*_-3_*z*_4 _ (54e)
*ż*_5 _= *k*_1_*E*_1 _(*z*_3 _- *z*_5_) - *k*_-1_*z*_5 _+ *k*_5_*E*_3 _(*z*_1 _- *z*_5_) - *k*_-5_*z*_5 _ (54f)
*ż*_*6 *_= *k*_2 _(*z*_3 _- *z*_5 _- *z*_6 _+ *z*_7_) *E*_2 _- *k*_-2 _(*z*_6 _- *z*_7_) + *k*_4 _(*z*_2 _- *z*_4 _- *z*_6 _+ *z*_7_) *E*_3 _- *k*_-4 _(*z*_6 _- *z*_7_) + *k*_3 _(*z*_5 _- *z*_7_) *E*_2 _+ *k*_5 _(*z*_4 _- *z*_7_) *E*_3 _- (*k*_-3 _+ *k*_-5_) *z*_7 _ (54g)
*ż*_7 _= *k*_1 _(*z*_6 _- *z*_7_) *E*_1 _- *k*_-1_*z*_7 _+ *k*_3 _(*z*_5 _- *z*_7_) *E*_3 _- *k*_-3_*z*_7 _+ *k*_5 _(*z*_4 _- *z*_7_) - *k*_-5_*z*_7 _ (54h)

**Table 5:** Kinetic parameters for dynamic simulation

Affinity of domain	*k*_*on *_[*M*^-1^*min*^-1^]	*k*_*off *_[*min*^-1^]	Equilibrium *K*_*d *_[*M*^-1^]
1 (always)	*k*_1 _= 3·10^5^	*k*_-1 _= 6	5·10^4^
2 (domain 1 unoccupied)	*k*_2 _= 1	*k*_-2 _= 18	5.6·10^-2^
2 (domain 1 occupied)	*k*_3 _= 5·10^7^	*k*_-3 _= 24	2.1·10^6^
3 (domain 1 unoccupied)	*k*_4 _= 1	*k*_-4 _= 12	8.3·10^-2^
3 (domain 1 occupied)	*k*_5 _= 1·10^5^	*k*_-5 _= 60	1.7·10^3^
